# Isotope Fingerprinting as a Backup for Modern Safety and Traceability Systems in the Animal-Derived Food Chain

**DOI:** 10.3390/molecules28114300

**Published:** 2023-05-24

**Authors:** Maria Olga Varrà, Emanuela Zanardi, Matteo Serra, Mauro Conter, Adriana Ianieri, Sergio Ghidini

**Affiliations:** 1 Department of Food and Drug, University of Parma, 43126 Parma, Italy; 2 Department of Veterinary Science, University of Parma, 43126 Parma, Italy

**Keywords:** fish, meat, milk, eggs, authentication, food fraud, IRMS

## Abstract

In recent years, due to the globalization of food trade and certified agro-food products, the authenticity and traceability of food have received increasing attention. As a result, opportunities for fraudulent practices arise, highlighting the need to protect consumers from economic and health damages. In this regard, specific analytical techniques have been optimized and implemented to support the integrity of the food chain, such as those targeting different isotopes and their ratios. This review article explores the scientific progress of the last decade in the study of the isotopic identity card of food of animal origin, provides the reader with an overview of its application, and focuses on whether the combination of isotopes with other markers increases confidence and robustness in food authenticity testing. To this purpose, a total of 135 studies analyzing fish and seafood, meat, eggs, milk, and dairy products, and aiming to examine the relation between isotopic ratios and the geographical provenance, feeding regime, production method, and seasonality were reviewed. Current trends and major research achievements in the field were discussed and commented on in detail, pointing out advantages and drawbacks typically associated with this analytical approach and arguing future improvements and changes that need to be made to recognize it as a standard and validated method for fraud mitigation and safety control in the sector of food of animal origin.

## 1. Introduction

In the past decades, there has been a substantial increase in the availability of food sources, thanks to improved food production and convenient cross-border transportation. As a result of the large supply and diversification of food products available on the market, consumers have become increasingly inclined towards choosing the ones that are associated with better quality attributes [1,2]. There are several interconnected reasons that explain why an increasing number of consumers prefer food products that have distinct characteristics, especially those that are strongly linked to a specific provenance. These reasons mainly relate to the perception of the safety of such products, as well as to their importance in social and cultural contexts [3]. In this setting, factors such as patriotism, skepticism towards products of unknown or non-native origin, the connection between locally produced goods and superior sensory qualities, and contemporary concerns regarding animal well-being and environmental sustainability in the food industry are of extreme importance [4,5]. Because of this, producers have gained significant market recognition and charged premium prices for products with distinctive geographic origins, while products of unknown origin cannot actually command the same level of value [6,7]. Consequently, the opportunities for abusers to take advantage of the economic gain from selling products marketed as high-quality but lacking the declared quality attributes have grown exponentially [8].

Over time, the food supply chain has been impacted by a range of food frauds, which include adulteration, counterfeiting, and imitation of popular brands, all with the goal of enhancing the perceived quality of products [2]. Nevertheless, this type of fraud has experienced a reduction in recent years, whereas there has been a dramatic rise in the mislabeling (absent, incomplete, or falsified labeling) of the country of origin and traceability issues within the supply chain of food of animal origin [9]. Indeed, according to the European Commission Knowledge Centre for Food Fraud and Quality, meat and seafood have been among the most defrauded items within the initial quarter of 2023 [10]. Almost all the fraudulent activities related to these products involved the lack of traceability documentation, with one major fraud incident in the UK meat sector involving the mislabeling of beef from South America and Europe as “best British beef” [10].

Nowadays, the determination of food authenticity and the strengthen of food traceability along the supply chain represent crucial issues for primary producers, the food industry, and food authorities, as demonstrated by the promulgation of the European Regulation EC No. 178/2002 [11], which makes traceability compulsory for all food and feed businesses, as well as by the Regulation (EU) 2017/625 [12], which laid the groundwork for a risk-based control of the authenticity of foodstuffs by the implementation of standardized and validated methods [8,13].

Over the years, many untargeted methods based on the fingerprinting or profiling of multiple organic or inorganic food components have been developed [14]. Stable isotopic ratio analysis, aimed at measuring the relative proportion of isotopes of both “light” elements (H, C, N, O, and S) and “heavy” elements (Sr, Cd, Pb, etc.), has emerged as one of the most promising techniques and has been used in food control since around the 1990s [15,16]. Recently, it has shown great potential in providing accurate information about the dietary and environmental background of animals, starting to be successfully applied to solve multiple authenticity issues, including the geographical origin and the production method of different food of animal origin such as milk, dairy, meat, and seafood [7].

Several advantages can justify the rise of isotope ratio analysis in food authentication studies, including its requirement for only a small sample amount and its high accuracy and robustness. Isotopic ratios are generally not altered during the manufacturing process, making the technique ideal for raw and processed foodstuffs as well [4,13]. However, it is important to note that foods of animal origin are typically characterized by a much more complex and varied isotopic profile compared to plant-based foods. This is because the isotopic abundances in animal tissues and secretions are affected by several interconnected factors such as their feeding habits, the trophic level, and the geoclimatic characteristics of their area of provenance [6,17]. Furthermore, different tissues of the same animal species and even different portions of the same tissue can vary widely in terms of isotopic composition from one another due to their unique physiological and metabolic functions and the different distribution of macromolecules such as lipids, proteins, and water (which each have their own isotopic composition) [6]. Although these factors offer valuable information for tracing the origin of animal-based food products, they can significantly complicate the interpretation of the final results or yield multiple possible interpretations.

To address these limitations, researchers have begun to merge stable isotope ratios of those elements mirroring the animals’ dietary background (such as C and N) with those more strongly associated with climatic and pedological factors (H, O, and S) or with data originating from other techniques. One strategy that has become increasingly prevalent involves combining stable isotope ratios of C and N with multi-elemental profiles, which provides complementary or synergistic information about the foodstuffs being studied and leads to improved accuracy and specificity in discriminating the sample even on the basis of complex authentication objective such as the geographical provenance [18,19].

The present review covers 135 research papers concerning the use of isotope fingerprinting as an authenticity and traceability tool for foods of animal origin, with a particular focus on factors influencing the isotopic ratio values of different elements in fish, meat, milk, and egg products. Advances in the field have been illustrated by analyzing from time-to-time both successful and unsuccessful current applications of the technique and by exploring the growing trend of incorporating isotopes with multiple markers. While the present review does not include a comprehensive explanation of the current instrumentations and methodologies used, interested readers can find more information by referring to other extensive literature reviews [20,21,22,23,24,25,26].

## 2. A General Picture of Trends and Tendencies in Using Isotopic Ratios to Trace Foods of Animal Origin

The 135 original research articles that make up the body of relevant literature considered in this review underwent a thorough analysis of trends in both temporal evolutions over the last decade (2010–2022) and covered topics. Detailed information concerning each article has been provided in Appendix A, where the literature, aggregated by food macro-category, has been summarized in the following tables: Table A1 (applications of isotope ratio analysis to fish and seafood), Table A2 (applications of isotope ratio analysis to meat and meat products), Table A3 (applications of isotope ratio analysis to milk and dairy), and Table A4 (applications of isotope ratio analysis to eggs).

**Figure 1:**
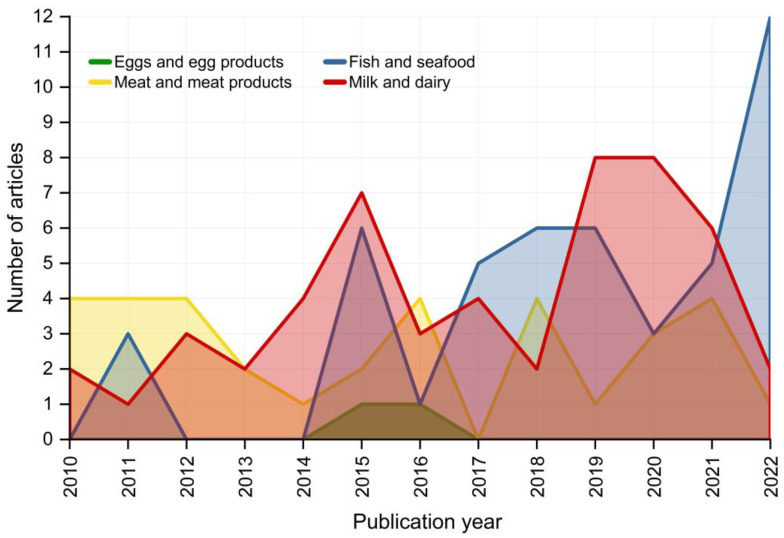
Trends over the last decade in research using isotope ratio analysis for authenticity and traceability of different animal-based food products.

Figure 1 depicts the frequency at which the isotope ratio analysis has been used to trace and authenticate animal-derived food in the past 12 years. Fluctuations in patterns depending on the specific food item being studied can be observed. Notably, interest in studying fish and seafood has substantially risen since 2015, with 12 research papers published on this topic in 2022 alone. On the other hand, research on meat and meat products has shown a more stable pattern until recently, with a slight increase in publications from 2019–2020, followed by a decline in 2021–2022 (Figure 1). Important research gaps were found for eggs, as they have only been studied twice in consecutive years and have not been the focus of any research for the past 5 years (Figure 1).

**Figure 2:**
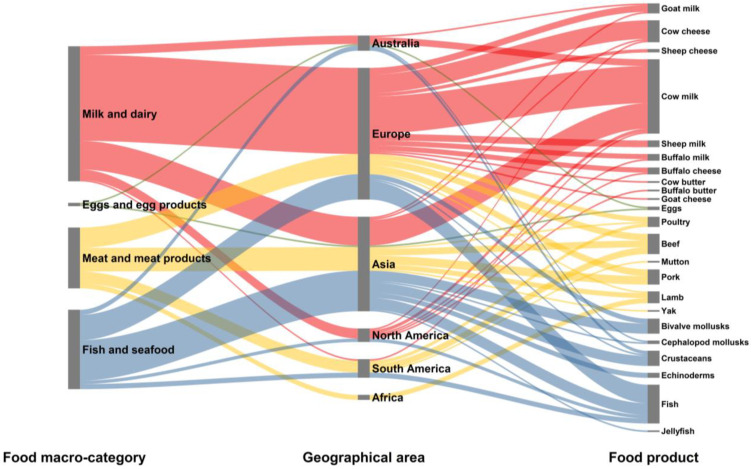
Alluvial plot depicting the frequency and patterns among food macro-categories, countries of study, and specific food items across scientific literature dealing with the use of isotope ratio analysis to assess authenticity and traceability of food of animal origin (the width of the nodes and flows corresponds to the amount of published data).

Figure 2 illustrates the proportion of the reviewed scientific literature across the different food macro-categories, countries, specific food products, and countries. The data indicates that Asian countries, followed by European ones, have been actively involved in studying the isotopic ratios of different elements in fish and seafood. On the other hand, research articles related to milk and dairy have been predominantly published in Europe (particularly in Italy, Spain, and Greece), followed by Asia, with China displaying an increasing interest in the field. As for meat and meat products, research efforts have been equally distributed between European and Asian countries, with significant contributions also from South American countries (especially Brazil and Chile). In Africa, measuring isotopic ratios for the traceability of meat was the only topic investigated, with a focus on authenticating lamb meat (as discussed in subsequent chapters). Unsurprisingly, these patterns were a clear reflection of the primary dietary habits, scientific expertise, and primary food production orientation of each country.

Further analysis revealed that cow milk has been the most frequently studied food item, followed by fish and cow cheese, while beef, pork, bivalve mollusks, and crustaceans have received similar levels of attention from researchers (Figure 2).

**Figure 3:**
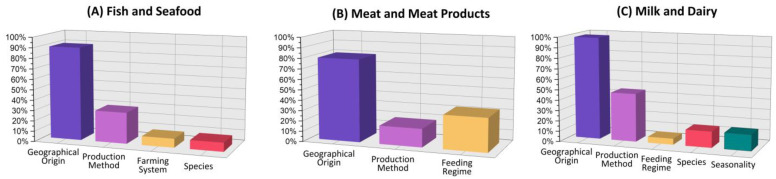
Distribution percentages of authenticity and traceability topics covered by the 2010–2022 scientific literature dealing with the use of isotope ration analysis for various animal-derived food products.

In particular, by analyzing in detail the 47 research articles dealing with fish and seafood, it emerged that fish has been the primary focus of the most recent literature (51% of the reviewed articles), followed by crustaceans and bivalve mollusks (20% each), and echinoderms (9%). Only two applications of isotope analysis were reported for cephalopod mollusks (Table A1 and Appendix A), and one application was made to certify the geographical origin and the production method of novel foods like jellyfish [27]. In addition, the majority of studies concentrated on fresh (unprocessed) fish and shellfish, with only three studies attempting to authenticate seafood after industrial transformations such as salting, smoking, and aging. The assessment of whether the original isotopic fingerprint of fresh products, connected to traceability and other authentication issues, is retained, or lost, during the transformation process is indeed the most challenging task. Globally, the assessment of geographic origin has been the authenticity topic that has received the most attention (89% of the reviewed articles), followed by the production method (30%), farming method, and biological species identification (both 9%) (Figure 3A and Table A1, Appendix A).

Among the 34 research papers on the use of isotopic analysis to assess the authenticity and traceability of meat and meat products (Table A2, Appendix A), 41% focused on bovine meat, 24% on sheep meat, 24% on pork, and 18% on poultry. Geographical origin authentication has been the main research purpose in 78% of the reviewed articles, while only 32% and 18% of the works dealt with the assessment of the feeding regime and production method, respectively (Figure 3B). Globally, the origin of beef, pork, and sheep meat was more often investigated than their production method or feeding regime, whereas the origin and production method of poultry received similar attention. No studies were found on biological species authentication (Table A2, Appendix A), likely due to the fact that utilizing DNA-based methods would be more effective and straightforward for this purpose.

With a total of 52 publications found, isotope ratio analysis to verify the authenticity of milk and dairy products was found to be the subject that has undergone the greatest research (Figure 1 and Figure 2). Ninety percent of research applications were focused on cow milk and cow dairy products due to their widespread dietary consumption, as well as their major use in the production of derived products with recognized quality marks such as the protected designation of origin (PDO) certification (Figure 3C and Table A3, Appendix A). One out of every two studies on non-bovine dairy products focused on sheep and goat cheeses, which require instruments for fraud protection due to their deeply ingrained regional identity and history (Table A3, Appendix A). Even for milk and dairy, more than 90% of the current research applications aim to discriminate samples based on the country of origin, while the production method and the feeding regime together were investigated in 52% of cases. The identification of the animal species from which milk and cheese were produced as well as the production season together represented 30% of the authentication topics.

## 3. Applications and Motivations of Stable Isotope Fingerprinting to Animal-Derived Foods

### 3.1. Fish and Seafood

It is well known that the isotopic composition of fish tissues is the result of several factors, such as the positions in food chains, the feed ingested throughout all life, kinetic fractionation due to metabolism, and geographical origin [17,28]. In particular, values of *δ*^13^C and *δ*^15^N in seafood are strongly influenced by the trophic position and feeding habits of each species since they vary according to the type and availability of prey and vegetations eaten by the animal, as well as the fractionation due to the metabolic processes that propagates along the aquatic food web [29,30,31]. Therefore, *δ*^13^C and *δ*^15^N measurements may be particularly useful to discriminate seafood according to the biological species, especially marine predators, which are at the top of the trophic chain. Taking advantage of this connection, Atlantic bluefin tuna (*Thunnus thynnus*), albacore (*Thunnus alalunga*), bullet tuna (*Auxis rochei*), and Atlantic mackerel (*Scomber scombrus*) were discriminated against each other by using *δ*^13^C and *δ*^15^N values, but it was necessary to include information derived from fatty acid composition to achieve satisfying results [32]. Similar to this, the integration of *δ*^13^C and *δ*^15^N with concentrations of toxic metals such as As, Cd, Hg, and Pb, has been confirmed as a powerful tool also for the accurate discrimination of eleven different freshwater fish species [33] and, at lower trophic levels, also of seven different shrimp species [34].

Since the diet composition of fish is unique to each living environment, values of *δ*^13^C and *δ*^15^N have also been used as markers for identifying the geographic origin of various fish species. The fact that *δ*^15^N may be influenced by local environmental parameters, such as water salinity and human inputs from agricultural runoff or wastewater treatment, lent more credence to this argument [33] to the point that the vastest body of literature has been focused on this authenticity topic (Figure 3A and Table A1, Appendix A). Additionally, from the reviewed research studies, it appeared that *δ*^13^C and *δ*^15^N are more informative of geographical origin than biological species, and they seem to work on any kind of seafood, including crustaceans, echinoderms, bivalve mollusks, and cephalopod mollusks (Table A1, Appendix A). It should be noted, however, that the resolving power of *δ*^13^C and *δ*^15^N alone was rarely reported as being high enough to perfectly discriminate the samples since the good results achieved were primarily the consequence of the additional information provided by the coupling with other biological indicators. Among these, poor attention has been paid to the coupling of *δ*^13^C and *δ*^15^N with the specific group of elements corresponding to rare earth elements, although these were reported as emerging authenticity indicators of food provenance [16]. Indeed, rare earth element profiles of fish tissues strongly reflect those of the local marine environment, which, in turn, is influenced by the geology of the underlying soil [16,28]. Rare earth element concentrations in the environment are also stable over years, and this makes them particularly attractive markers of geographical origin. Within this framework, we previously demonstrated an important contribution of *δ*^13^C, *δ*^15^N, La, and Ho in the discrimination of sea bass samples from three neighboring fishing areas located in the Mediterranean Sea [28].

Compared to *δ*^13^C and *δ*^15^N, the isotopic abundances of H, O, and S (*δ*^2^H, *δ*^18^O, and *δ*^34^S) in fish tissues have a more direct relationship with the geographical provenance. As a matter of fact, the tissues of animals assimilate H atoms from the surrounding water and their diet, with a gradual enrichment in ^2^H along the aquatic food chain [35]. In contrast, *δ*^34^S is associated with the biogeochemical sulfur cycles and does not fractionate within the aquatic food chain [36]. The values of *δ*^18^O in seawater depend on the salinity, the composition of the water mass, and the rate of surface evaporation, with enrichment rising steadily from higher to lower latitudes [37]. Despite this link, only a small number of studies have evaluated the *δ*^2^H, *δ*^18^O, and *δ*^34^S isotopic fingerprints for the verification of the geographical origin of mussels [38], clams [36,39], sea bass [30], and seawater/freshwater salmonids [40,41]. In this setting, it emerged that these isotopic signatures were effective in revealing not only the geographical provenance, but also the production method of the products [40,41].

It is important to note that, at present, assessing the geographical origin of fish and seafood is a complex undertaking, fraught with numerous challenges, particularly when investigating species with intricate life histories and ecological roles, such as top predators and anadromous species [42]. These species undergo significant changes in their feeding habits and habitats throughout their lives, transitioning to environments with distinct isotopic characteristics. Consequently, prior to reaching an equilibrium state with the new system, their tissues may contain a very complex isotopic signature, leading to significant challenges in interpreting and accurately determining their geographical origin [6,42]. Within this context, a potential groundbreaking approach, called the “isotopic clock” [43], holds promise for overcoming these complexities. The isotopic clock method relies on the principle that different tissues within the same specimen have varying metabolic turnover rates, which means they adjust to dietary isotopic changes at different speeds [43,44]. Consequently, by examining isotopic signatures from multiple tissues of fish and developing a calibrated model based on the timing of environmental or dietary shifts, it may become possible to overcome factors that complicate the identification of the geographical origin. The isotopic clock approach holds significant potential in enhancing the precision of tracking particular fish species, underscoring the need for extensive research into its application within the field of food authentication.

Interestingly, Chen et al. [45] and Thomatou et al. [46] analyzed the stable isotopic composition of the highly valuable mullet (Mugilidae fish family) roes, which are produced from salt-drying of mullet eggs. Despite the products being processed, the authors verified that isotopic information of the raw material was maintained, allowing for the identification of the final products by their geographical origin and production method.

Probably due to analytical issues, the evaluation of higher mass isotopes is not common in literary works, and it is mainly limited to the evaluation of Sr (^87^Sr/^86^Sr) and Nd (^143^Nd/^144^Nd) ratios. In particular, Nd isotopes are a great promise for seafood traceability since they are not subjected to fractionation along the trophic chain and exhibit a diverse pattern based on provenances, thus being optimal candidates as unique tracers of origin [36]. The usefulness of ^87^Sr/^86^Sr and ^143^Nd/^144^Nd has been proven so far only for bivalves like mussels [47] and clams [36,39,48].

Finally, from the study conducted by Won et al. [36] and other studies focused on the origin traceability of bivalves [49,50,51], an oddity that emerged was the choice of the adductor muscle as the subject of isotopic analysis. This strategy deserves to be further explored in the future, because the adductor muscle, as opposed to the mantle, gonads, or digestive glands, is reported to have a slower turnover and a lower lipid content, thus better representing the long-term metabolic status of the mollusks [51,52].

### 3.2. Meat and Meat Products

Isotopic ratios of C and N in animal tissues have a close connection to the type of feed and water that the animal consumes during its life. In fact, fodder plants characterized by C3 photosynthetic pathways (rice, wheat, soybeans, rye, barley, potatoes, sugar beet, and common grass in temperate zones) have greater negative *δ*^13^C values than CAM plants (succulents) and C4 plants (maize, sorghum, sugarcane, and common grass in tropical zones) [53]. The carbon isotopic signature is therefore particularly useful for distinguishing the feeding patterns of livestock and pinpointing the geographic origin of animals fed on local grass [7,53]. This evidence served as the foundation for the findings of Rhodes et al., who were able to verify the authenticity of poultry labeled as corn-fed by relying solely on *δ*^13^C as a reliable marker of the animal’s dietary status [54]. Meat from lambs that were fed legume-rich diets was also identified with an accuracy of 88% due to the decreasing *δ*^15^N values that were observed as the proportion of alfalfa administered to the animals increased [55]. In addition, the combined use of *δ*^13^C and *δ*^15^N was effective in identifying the geographical origin of meat. For instance, when examining beef burgers from a multinational fast-food chain and distributed in 26 countries around the world, *δ*^13^C and *δ*^15^N values were found to range from 25.4‰ to 11.1‰ and 5.9‰ to 7.3‰, respectively, with samples from higher latitude countries showing lower *δ*^13^C values than those from lower latitude countries [56]. These findings suggested that this method can be useful for verifying the authenticity of even highly processed food products, but only when applied to samples collected from geographically distant locations and mainly due to the contribution of *δ*^13^C. Indeed, when attempting to identify the origin of meat on a smaller scale (such as different regions within the same country) and using the sole *δ*^15^N indicator, the same method was unsuccessful [57,58].

To address authenticity concerns about geographical origin, some authors measured isotopic ratios of H, C, N, O, and S simultaneously. The *δ*^2^H and *δ*^18^O in animal tissues are influenced not only by the diet, but are also directly subjected to strong seasonal effects, as well as geographic latitude and altitude effects. H and O are indeed mainly sourced by the animal from the drinking water, whose isotopic composition, in turn, derives mainly from those of precipitation and subsoil waters [59,60,61]. Indeed, it has been reported that precipitation waters fallen during the winter season, and at increasing latitude and altitude, tend to show more negative *δ*^2^H and *δ*^18^O values [62]. The S isotopic signatures of animal tissues have a stronger link with the characteristics of the soils where the plants used as feed were grown, and vary according to natural mineral compositions, soil microbial activities, and distance from the sea [63,64,65].

Using the complementary information provided by all the light stable isotope ratios, it was possible to distinguish beef from nine different European and non-European countries with an accuracy of 82%, as well as to achieve a perfect accuracy of 100% for identifying Irish pasture-fed beef [66]. Additionally, the use of all light stable isotope ratios enabled the small-scale classification of lamb samples from distinct regional farming systems in Tunisia with an overall accuracy of 94%. Of all the isotopic ratios, *δ*^34^S was the most effective in distinguishing lamb types, as it served as a clear “coastal” signal due to the influence of the so-called sea-spray effect [65].

Isotope ratio analysis has useful and compelling applications also for tracing dry-cured hams. According to Perini et al., the isotopic variations observed between Italian protected designation of origin (PDO) and Spanish hams can be mainly ascribed to the isotopic makeup of meteoric water and the amount of C4 plants in the animals’ diet. However, the researchers also observed that the different duration of the ripening–drying phase of the tested products resulted in different O enrichments and S and H depletions in the protein fraction, along with H enrichments in the marbling fat. This result suggests that a non-negligible impact of the processing method on the final isotopic profiles of the products may actually exist [67]. Interestingly, ^87^Sr/^86^Sr can also be considered a useful parameter to trace back European hams from Spain, Portugal, France, and Italy, where the ^87^Sr/^86^Sr composition of the salt used in the manufacturing process seems to have a crucial role [68]. However, despite the valuable insights that can be gained about the soil properties of the studied area from the ^87^Sr/^86^Sr ratio, this indicator is not commonly utilized, likely due to the requirement for costly, specialized equipment and extensively trained staff [69].

Out of all animal-based foods that were studied, only meat and meat products underwent analysis of a wide range of isotopes of heavier elements for traceability purposes, but examples remain confined to only a handful of applications (Table A2, Appendix A). Based on the hypothesis that the Pb isotope signature of livestock could indicate the interaction of the animal with the environment and that Pb fractionation due to biological processes is negligible [4], British mutton, cow, and chicken samples were authenticated by measuring the four naturally occurring Pb isotopes and five of their ratios (^206^Pb/^204^Pb, ^207^Pb/^204^Pb, ^208^Pb/^204^Pb, ^207^Pb/^206^Pb, ^208^Pb/^206^Pb) [70]. The authors ruled out the impact of tetraethyl anthropogenic Pb and confirmed that the Pb isotopic signature in the animal muscle was due to geogenic exposure [70]. Additionally, several isotopic ratios of other elements—including Ag, B, Cd, Cr, Cu, Ga, Li, Ni, Mo, Sr, and Zn—were identified as useful tracers for determining the origin of pork meat and pork belly fat from various countries [71,72]. The encouraging outcomes imply that the novel approach of measuring ratios of stables or radiogenic isotopes of heavier elements warrants an additional exploration in the coming years and deserves to be applied to other animal-derived foods to establish innovative means for verifying their provenance.

Finally, it should be emphasized that the variation in isotope ratios within different muscle and tissue types of the same animal had not been adequately addressed in previous studies. This lack of understanding could pose a challenge to accurately interpreting isotopic profiles and making direct comparisons between samples. Therefore, further research on this subject should be greatly encouraged.

### 3.3. Milk and Dairy Products

One noteworthy difference between the isotope research on milk and meat or fish is that in the former, there has been a greater emphasis on measuring isotopic ratios on isolated macro-fractions (e.g., lipids, water, casein, and whey) or even individual molecules (single fatty acids and amino acids) using the so-called compound-specific isotope analysis (Table A3, Appendix A). This approach is often preferred over bulk isotope analysis of a whole sample because it eliminates variations in stable isotope ratios that naturally exist between different fractions of the sample. To give an illustration, analyzing the bulk isotopic composition of milk from different sources of the same type may yield inaccurate results in terms of *δ*^13^C values, since protein is significantly enriched in ^13^C compared to lipids. As a result, due to compensation effects arising from natural variations in the protein and lipid content, the *δ*^13^C values may not show significant differences related to sample origin among the milk samples, even though the differences actually exist. Consequently, to ensure the reliable and consistent classification of samples for a specific authentication purpose, it is better to analyze the isotopic values of either the lipid or protein fraction, as this would yield more consistent and comparable results. The majority of the studies mentioned below analyzed the stable isotope ratios of separated components, such as casein, glycerol, lactose, fat, whey, water, or glycerol, in both milk and cheese samples.

As for meat, carbon isotopes in milk are mainly related to the specific C3, CAM, or C4 photosynthetic pathways of the plant supplied as feed [20,73]. The *δ*^13^C values of plants tend to be reflected in milk, thus providing direct information on the specific husbandry practices and production method used for breeding, but also indirect information about the geographical origin (provided that cattle are fed with local feedstuffs) [74,75,76]. For instance, *δ*^13^C alone allowed for the differentiation of organic cow and buffalo milk from conventional milk, providing the advantage of not being fractionated throughout the production process and, therefore, being useful also as a marker for organic cheeses [74,75,76]. Since *δ*^15^N values are typically lower in milk from cows fed a high concentration of feed and raised on synthetic fertilizer-treated soils [77], *δ*^15^N has also demonstrated good potential as an authenticity marker of organic dairy [78,79,80,81], to the point that a threshold value of *δ*^15^N ≤ 5.5‰ in the defatted dry matter to uniquely identify organic productions has been proposed [81]. By combining the chemical information enclosed within *δ*^13^C and *δ*^15^N values of milk produced in a specific geographic region from animals following a well-defined diet, the possibility of discriminating between high-value PDO cheeses and non-PDO cheeses was also proved [76,82,83,84]. Scampicchio et al. are credited with one final interesting use of the C and N isotopic signatures to verify milk in accordance with the manufacturing process [85]. The authors stated that the technological processing, such as the conventional heat processing, is responsible for the decrease of *δ*^15^N values in the whey fraction and their increase in the fat fraction of pasteurized and ultrahigh-temperature processed milk, making it feasible to use this information to detect the treatments applied to commercial milk [85].

Sources of H and O isotopes in milk are the same as those reported for meat, being strongly related to the isotopic composition of drinking water and, in turn, varying according to seasonality, latitude, and altitude [59,60,62]. Evidence of the transfer of the isotopic composition of meteoric waters to milk was provided by Behkami et al., according to whom a strong positive correlation between *δ*^2^H and *δ*^18^O values of cow milk with those of the rain exists [86]. The authors also found that the latitude of the sampling site can also affect the C and N isotopic distribution of the milk to the point that, when coupled with *δ*^2^H and *δ*^18^O, milk samples can be discriminated based on their geographical origin.

The effect of altitude on δ *δ*^2^H and *δ*^18^O dairy composition has also been successfully exploited to authenticate different mountain cheeses according to the altitude of the alpine environment [87]. On the other side, *δ*^18^O values in animal secretions also vary depending on the species. This variation results from the physiological water balance of the animal and is caused by evapotranspiration, which determines different water isotope fractionations in body fluids [59,88,89]. As a result, cow milk was found to have lower *δ*^18^O values than sheep and goat milk [59], opening possibilities for its use in the future to identify milk adulteration by mixing milk from different animal species.

The Sr isotope composition of milk has been linked to the geological background of its origin and the drinking water supplied to the animal [90,91]. This composition remains unchanged even after food processing [92], making it an effective way to trace country-specific information, even in highly processed foods like infant formula milk powder [93]. However, only four recent studies dealing with the measurement of ^87^Sr/^86^Sr in dairy were found, and these studies mainly attributed their results to the combination of Sr with other isotopic and/or elemental markers through chemometrics [77,90,93,94]. For instance, the possibility of discriminating with high specificity and accuracy between artisanal and commercial goat cheeses from Canada and Europe was achieved through a combination of the climate-sensitive *δ*^18^O and geology-sensitive ^87^Sr/^86^Sr values [77].

As discussed in previous chapters, S isotopes are strongly related to the geological characteristics of the soils. Nevertheless, relying solely on *δ*^34^S values to determine the specific geographical origin of animal-derived food might not be dependable because the feeding patterns can create overlapping and confusing isotopic fluctuations, even in milk. This is exemplified by the fact that milk from cows receiving the most concentrated diet had an average *δ*^34^S value of 5.47‰, while milk from cows fed mostly grass had an average *δ*^34^S value of 6.62‰ [95]. Notwithstanding this limitation, several attempts at using *δ*^34^S value to identify the country of origin of milk and dairy products from different animal species have been made. However, it is significant to note that the authors did not attribute the positive results solely to S as a geographic determinant. Instead, they mainly based their conclusions on the combined analysis of other stable isotope ratios and/or multi-element profiles (Table A3, Appendix A), where *δ*^34^S had no impact [96,97] or little bearing on the geographical discrimination [84,98,99].

The above description and Table A3 (Appendix A) suggest that relying solely on stable isotope ratio analysis may not always provide satisfactory results in terms of discrimination power due to the possible overlaps of isotopic signatures among different geographical areas or production systems. Therefore, the integration of multiple analytical techniques was often recommended to improve the reliability and accuracy of milk authenticity control. In addition to elements and fatty acids, other markers have been proposed to enhance the accuracy of milk authenticity discrimination. Within this context, it was demonstrated that the fusion of data from stable isotope ratio analysis with multielement fingerprinting and fatty acid profiling can allow for the discrimination of milk samples from Australia, New Zealand, and Austria, with optimal results achieved when considering *δ*^15^N, *δ*^18^O, As, Ba, Ca, Cs, Eu, K, Mo, Rb, Sc, Sr, Tl, C20:4n6, C13:0, and C16:1n7 simultaneously [100]. Similarly, a study conducted by Xie et al. suggested that a combination of nutritional parameters (amino acids) and geographical parameters (stable isotopes and elemental analysis) is the best choice to distinguish milk from very small-scale regions [101]. Based on the same principle, Erich et al. demonstrated that combining the outputs of various data sources, including NMR spectroscopy, stable isotope ratios, fatty acid profiles, and α-linolenic acid content, can provide a more efficient differentiation between conventional and organic milk [102].

### 3.4. Eggs and Egg Products

Researchers have shown very little concern for verifying the authenticity and traceability of eggs (Table A4, Appendix A), even though these products are highly susceptible to fraud because of false claims about their production methods, such as those declaring organic, free-range, or non-GMO feed production methods.

Rogers et al. monitored C and N isotopic distributions in egg whites from The Netherlands and New Zealand for over seven years in order to discriminate between conventional and organic egg farming systems. The authors found that both organic and conventional eggs from The Netherlands were characterized by lower *δ*^15^N values than those from New Zealand, because in the former no fishmeal or meat and bone meal were used. Based on this result, they suggested establishing critical *δ*^15^N values to certify organic eggs of at least 4.8‰ and 6.0‰ for Dutch and New Zealand products, respectively [103].

The possibility of discriminating the pigment type added to poultry feedstuffs and transferred to eggs was also investigated through the determination of *δ*^13^C and *δ*^15^N. An enrichment of *δ*^13^C values of the yolk was observed only with increasing maize content in the diet, while the addition of carophyll red, carophyll yellow, or a combination of both pigments had no impact [104].

In summary, due to the limited availability of isotopic data on eggs, it is crucial to collect more information on this subject. Therefore, it is highly recommended to conduct further investigations to fill this knowledge gap and enhance the understanding of their applicability for traceability purposes.

## 4. Charting Future Research Directions

As emerged from the reviewed body of literature, stable isotopes, especially those of light elements and, to a lesser extent, heavier elements, can be considered a powerful biological, ecological, and geochemical indicator of several quality features of food of animal origin [36].

Probably the most significant benefit of using isotopic ratios is their consolidation within the international scientific community and its good standardization by international organizations, which can ensure reliable and consistent results and make it possible for regulatory agencies to adopt them as an official method for food authenticity and traceability assessment. On the other hand, the high costs of the available equipment make the method only accessible to a few advanced research laboratories and almost non-existent in routine analysis laboratories. This inevitably results in a reduction in the volume of data generated and in a slowdown in the advances of knowledge in the field.

Research applications of stable isotope ratio analysis for multi-purpose authenticity testing suggest that the method tends to show better performances for origin discrimination rather than species or production method/farming system discrimination. However, this outcome can be interpreted with greater confidence only when the discrimination by geographical origin was addressed to wild animals, and, therefore, it is mainly applicable to fish and seafood. Indeed, isotopic ratios can have poor discrimination power or lead to inconclusive results when used alone to assess the geographical provenance of meat, milk, and eggs. Since these products derive from livestock animals that have potentially received the same internationally marketed feeds, any isotopic differences that would indicate their origin may be masked or indistinguishable [18].

To avoid drawing biased conclusions regarding the efficacy of stable isotopes in identifying the source of origin, it is also necessary to clarify that in numerous instances, distinguishing between fish, meat, and milk in research has been accomplished through a combination of stable isotope ratios and profiles of multi-elemental profiles. Although using multiple discriminatory variables may seem advantageous, the flip side is that it can be challenging to implement these techniques in routine analysis due to the expensive instrumental equipment, the lengthy analysis times, and the complexity of applying suitable chemometric methods to process and merge vast and diverse analytical data. To partially overcome these limitations, a highly effective and practical approach would be to narrow down the field of analysis and solely concentrate on the combination of variables that possess the strongest discriminatory power. This approach would optimize both the technical and economic aspects of the entire workflow, while retaining or even increasing the accuracy and sensitivity of the measurements thanks to the elimination of noisy and redundant data. In this context, merging stable isotope ratios of light elements with a limited group of elements (such as rare earth elements) or isotope profiles of high-mass elements (such as Pb isotopes), measured all together by analytical systems able to provide in situ and spatially resolved information, may represent an important step forward.

Research on isotopic tracers in the near future is expected to provide various benefits and challenges. However, it is crucial and urgent for researchers and interested parties to collaborate in creating comprehensive reference databanks collecting isotopic and elemental maps of food. Despite that ensuring thorough coverage and continued maintenance over time is a difficult task [105], reference databanks would represent a significant breakthrough in combating food fraud and ensuring food safety and authenticity.

## 5. Materials and Methods

The search strategy adopted to gather and analyze the relevant literature for this review article included the collection of articles from *ScienceDirect* and *Web of Science* databases, limiting the search to sources published between 2010 and 2022. The search terms that were used included “fish”, “seafood”, “crustaceans”, “mollusks”, “shellfish”, “meat”, “animal by-products”, “offal”, “pig”, “pork”, “calves”, “bovine”, “cattle”, “poultry”, “chicken”, “ovine”, “caprine”, “lamb”, “goat”, “milk”, “dairy”, “cheese”, or “eggs”, coupled with “food fraud”, “authenticity”, “traceability”, “geographical origin”, “farming system”, “production method”, “discrimination”, or “characterization”, and with “isotopes”, ‘light element isotopes”, “heavy element isotopes”, “isotope ratios”, or “isotopic fingerprint”. Initially, the search encompassed honey as well. However, upon review, it was decided to remove this food item from consideration. This decision was made primarily because the main literature focus was on evaluating adulteration rather than aspects related to authentication by geographical origin, production method, and traceability. Each article was screened for its relevance to the topic of this review by examining the title and the abstract. A total of 135 relevant sources were therefore selected. Full texts were then downloaded, and important data were extracted and summarized in a standardized data form, in which information concerning the authors, publication year, country, food macro-category, specific food product, analyzed tissue, sample size, measure isotopes, and other measure markers were annotated. Based on this information, specific research trends and patterns were identified and summarized in Table A1, Table A2, Table A3 and Table A4 of Appendix A. A narrative synthesis of the findings was conducted through the main text by reporting the most relevant research articles.

## 6. Conclusions

A wide range of environmental and biological factors affect the isotope abundances of light and heavier elements in animal tissues and secretions, leading to a unique fingerprint that can be used to identify food frauds affecting the animal-derived food chain. Following the current research developments in the field, the geographical origin, the animal diet, and the production system (organic/conventional, wild/farmed) of a variety of animal food products such as milk and dairy, meat, fish and seafood, and eggs, can be identified by using stable isotopic ratios of light elements. Nevertheless, the combination with other inorganic markers seems to be necessary to increase robustness in contrasting confounding results.

It is expected that advances in analytical technologies and big data handling would help the creation of comprehensive isotopic maps of foods, whose dissemination through comprehensive databanks would mark a significant milestone in modern animal-derived food traceability systems. This would improve the efficiency of food inspection and control procedures, assure a higher food safety standard, enhance transparency and regulatory compliance of foodstuffs, and, finally, contribute to preserving the integrity of the food supply chain.

## Data Availability

Not applicable.

## Appendix A

**Table A1:** Works published within 2010–2022 dealing with the authenticity verification of fish and seafood using isotope ratio analysis.

Product	Tissue	Purpose *	Isotopes	Other Markers	Ref.
*Cnidarian*					
Flame jellyfish (*Rhopilema esculentum*), Nomura’s jellyfish (*Nemopilema nomurai*)	Bell tissues	GO, PM	*δ*^13^C, *δ*^15^N	Fatty acids	[27]
*Crustaceans*					
Swimming crab (*Portunus trituberculatus*)	Gills, claw, hepatopancreas, abdominal muscle	GO	*δ*^13^C, *δ*^15^N	Na, Mg, Al, K, Mn, Fe, Co, Cu, Zn, As, Se, Rb, Ag, Ba	[31]
Louisiana crawfish (*Procambarus clarkii*)	Abdominal muscle	GO	*δ*^13^C, *δ*^15^N	Na, Mg, Al, K, Ca, Mn, Zn, Cu, Fe, Sr, Ba, As, Se, Cd	[106]
Pacific white shrimp (*Litopenaeus vannamei*)	Muscle	PM, FR	*δ*^13^C, *δ*^15^N	-	[107]
Black tiger prawn (*Penaeus monodon*)	Abdominal muscle	GO, PM	*δ*^13^C, *δ*^15^N	Mg, Al, Si, P, S, Cl, K, Ca, Ti, Cr, Mn, Fe, Ni, Cu, Zn, As, Se, Br, Rb, Sr, Y, Zr, Cd, Sn, Sb, Nd, Hf, Pb, Bi, At, U	[108]
Chinese mitten crab (*Eriocheir sinensis*)	Muscle	GO	*δ*^13^C, *δ*^15^N	Na, Mg, Al, K, Ca, Mn, Cu, Zn, Sr, Ba	[109]
Pacific white shrimp (*Litopenaeus vannamei*)	Muscle	GO	*δ*^13^C, *δ*^15^N	-	[110]
Shrimp (*Penaeus monodon*, *Litopenaeus vannamei, Fenneropenaeus indicus*, *Fenneropenaeus merguiensis*, *Farfantepenaeus notialis*, *Pleoticus muelleri*, *Pandalus borealis*)	Muscle	GO, PM, SP	*δ*^13^C, *δ*^15^N	Pb, Cd, As, P, S	[34]
Shrimp (*Pandalus borealis*, *Marsupenaeus japonicus*, *Fenneropenaeus chinensis*, *Litopenaeus vannamei*, *Penaeus monodon*, *Solenocera crassicornis*)	Muscle	GO	*δ*^13^C, *δ*^15^N	Cytochrome oxidase I (COI)	[111]
Tiger prawn (*Penaeidae family*)	Muscle, chitin, recovered water	GO	*δ*^2^H, *δ*^13^C *δ*^15^N, *δ*^18^O	Li, B, Al, Ti, V, Mo, Cr, Mn, Fe, Co, Ni, Cu, Zn, As, Se, Sr, Cd, Hg, K	[112]
*Echinoderms*					
Sea urchin (*Mesocentrotus nudus*)	Gonads, spines	GO	*δ*^13^C *δ*^15^N, *δ*^18^O		[113]
Sea cucumber (*Apostichopus japonicus*)	Body wall	GO	*δ*^13^C, *δ*^15^N	Ba, Li, Ca, K, Na, Mg, Al, Fe, Mn, Cu, Zn, Se, Cr, Co, Ni, As, Sr, Cd, Pb, Sn, V, Ag	[114]
Sea cucumber (*Apostichopus japonicus*)	Body wall	GO, PM	*δ*^13^C	Amino acids	[115]
Sea cucumber (*Apostichopus japonicus*)	Body wall	GO	*δ*^13^C, *δ*^15^N	Fatty acids	[116]
*Fish*					
Flathead grey mullet (*Mugil cephalus*)—salted/dried	Roes (ovaries)	GO	*δ*^13^C, *δ*^15^N *δ*^34^S	-	[46]
Silverfish (*Trachinotus ovatus*) and silver pomfret (*Pampus argenteus*)	Muscle	GO	*δ*^13^C, *δ*^15^N	K, Ca, Na, Al, Ti, Mn, Fe, Cu, Zn, Se, Rb, Sr, and Sn	[117]
Flathead grey mullet (*Mugil cephalus*)—raw, salted/dried	Roes (ovaries)	GO, PM, SP	*δ*^13^C, *δ*^15^N	DNA/RNA	[45]
Atlantic salmon (*Salmo salar*), rainbow trout (*Oncorhynchus mykiss*)	Muscle	GO, PM	*δ*^2^H, *δ*^18^O	-	[41]
Atlantic bluefin tuna (*Thunnus thynnus*), albacore (*Thunnus alalunga*), bullet tuna (*Auxis rochei*), Atlantic mackerel (*Scomber scombrus*)	Muscle	GO, SP	*δ*^13^C, *δ*^15^N	Mitochondrial DNA, fatty acids	[32]
European sea bass (*Dicentrarchus labrax*)	Muscle	GO, FS	*δ*^2^H, *δ*^13^C, *δ*^15^N, *δ*^18^O	-	[30]
Black carp (*Mylopharyngodon piceus*), silver carp (*Hypophthalmichthys molitrix*), grass carp (*Ctenopharyngodon idella*), common carp (*Cyprinus carpio*)	Muscle	PM, FS	*δ*^13^C, *δ*^15^N	Li, Be, Na, Mg, Al, K, Ca, V, Cr, Mn, Fe, Co, Ni, Cu, Zn, Ga, Rb, Sr, Mo, Ag, Cd, In, Cs, Ba, Tl, Pb, Bi	[118]
European sea bass (*Dicentrarchus labrax*)	Muscle	GO, PM	*δ*^13^C, *δ*^15^N	La, Eu, Ho, Er, Lu, Tb	[28]
European eel (*Anguilla anguilla*)	Muscle	GO, FS, FR	*δ*^13^C, *δ*^15^N	Fatty acids	[119]
Crucian carp (*Carassius carassius*), snakehead (*Channa argus*), carp (*Cyprinus carpio*), silver carp (*Hypophthalmichthys molitrix*), weever (*Lateolabrax japonicus*), redeye mullet (*Liza haematocheila*), wuchang (*Megalobrama amblycephala*), flathead grey mullet (*Mugil cephalus*), yellow catfish (*Pelteobagrus fulvidraco*), catfish (*Silurus asotus*), javelin gobi (*Synechogobius hasta*)	Muscle	SP	*δ*^13^C, *δ*^15^N	As, Hg, V	[33]
Atlantic salmon (*Salmo salar*) Pacific salmon (*Oncorhynchus gorbuscha*, *Oncorhynchus nerka*)	Muscle	GO, PM, FR	*δ*^13^C, *δ*^15^N	Phe, Lys, Leu, His, Gly, Asx, Ser	[120]
Rainbow trout (*Oncorhynchus mykiss*)	Muscle	GO, FR	*δ*^2^H, *δ*^13^C, *δ*^15^N, *δ*^18^O, *δ*^34^S	-	[40]
European sea bass (*Dicentrarchus labrax*)	Muscle	GO, PM, FS	*δ*^13^C, *δ*^15^N	Biometric traits, fatty acids, elements	[121]
Hake (Merlucciidae family)	Muscle	GO	*δ*^13^C, *δ*^15^N	-	[122]
Largemouth bass (*Micropterus salmoides*)	Muscle, liver	FR	*δ*^2^H, *δ*^18^O	-	[123]
Meagre (*Argyrosomus regius*)	Muscle	PM	*δ*^13^C, *δ*^15^N	Moisture, fatty acids, S, Cl, K, Ca, Fe, Zn, As, Se, Br, Sr	[124]
European sea bass (*Dicentrarchus labrax*)	Collagen (scales)	GO	*δ*^13^C, *δ*^15^N	-	[125]
Atlantic salmon (*Salmo salar*), Pacific salmon (*Oncorhynchus nerka*), Brown trout (*Salmo trutta*)*—* Raw, smoked, graved	Muscle	PM	*δ*^13^C, *δ*^15^N	Lipid content, carotenoids	[126]
Croaker (*Micropogonias furnieri*)	Muscle	GO, SEAS	*δ*^13^C, *δ*^15^N	Moisture, protein content, lipid content, S, Cl, K, Ca, Fe, Zn, As, Se, Br, Sr, Hg	[127]
Mackerel (*Scomber japonicas*), Yellow Croaker (*Larimichthys* *polyactis, Larimichthys crocea*), pollock (*Theragra chalcogramma*)	Muscle	GO	*δ*^13^C, *δ*^15^N	-	[128]
Hairtail (*Trichiurus japonicus, Trichiurus lepturus*)	Muscle	GO	*δ*^13^C, *δ*^15^N	Cytochrome oxidase I (COI)	[111]
Atlantic cod (*Gadus morhua*) *, saithe (*Pollachius virens*)*—*salted	Muscle, bone, skin	SP	*δ*^13^C, *δ*^15^N	-	[129]
Sea bream (*Sparus aurata*)	Muscle	GO	*δ*^13^C, *δ*^15^N	RNA, DNA, cytochrome-c-oxidase, citrate synthase,	[130]
Cachara (*Pseudoplatystoma fasciatum*)	Muscle	PM, SEAS	*δ*^13^C, *δ*^15^N	Protein content, lipid content, ash, moisture	[131]
*Mollusks*					
Manila clams (*Ruditapes philippinarum*)	Soft tissue, shell	GO	*δ*^13^C, *δ*^15^N, *δ*^18^O *δ*^34^S, ^87^Sr/^86^Sr,	-	[39]
Manila clams (*Ruditapes philippinarum*)	Adductor muscle	GO	*δ*^13^C, *δ*^15^N	Fatty acids	[51]
Manila clams (*Ruditapes philippinarum*)	Soft tissue	GO	^143^Nd/^144^Nd	-	[48]
Mediterranean mussel (*Mytilus galloprovincialis*)	Soft tissue, shell	GO	*δ*^13^C, *δ*^15^N	B, Al, Ti, V, Cr, Mn, Fe, Co, Ni, Cu, Zn, As, Se, Cd, Ba, Pb	[29]
Mussels (*Mytilus edulis*)	Soft tissue	GO	*δ*^2^H, *δ*^13^C, *δ*^15^N, *δ*^18^O	-	[38]
Manila clam (*Ruditapes philippinarum*)	Soft tissue, shell	GO	*δ*^13^C, *δ*^15^N	-	[132]
Manila Clams (*Ruditapes philippinarum*)	Adductor muscle	GO	*δ*^2^H, *δ*^13^C, *δ*^15^N, *δ*^18^O *δ*^34^S, ^87^Sr/^86^Sr, ^143^Nd/^144^Nd	-	[36]
Mussels (*Mytilus* spp.)	Shell	GO	^143^Nd/^144^Nd	-	[47]
Yesso scallop (*Patinopecten yessoensis*)	Adductor muscle	GO	*δ*^13^C, *δ*^15^N	Ala, Gly, Val, Leu, Ser, Phe, Fuc, Rha, Glc, Man	[50]
Scallops (*Patinopecten yessoensis*, *Chlamys farreri*, *Argopecten irradians*)	Adductor muscle	GO	*δ*^13^C	Fatty acids	[133]
Octopus (*Octopus berrima*, *Octopus pallidus*, *Octopus pallidus*, *Amphioctopus aegina*)	Soft tissue	GO	*δ*^13^C, *δ*^18^O	Al, Si, P, S, Cl, K, Ca, Ti, V, Cr, Mn, Fe, Ni, Cu, Zn, As, Br, Rb, Sr, Y, Zr, Sb, Ba, Ce, Sm, Pb	[134]
Jumbo squid (*Dosidicus gigas*)	Soft tissue, cartilage	GO	*δ*^13^C, *δ*^15^N	Fatty acids	[135]

* GO = geographical origin; FR = feeding regime; FS = farming system; PM = production method; SEAS = seasonality; SP = species.

**Table A2:** Works published within 2010–2022 dealing with the authenticity verification of meat and meat products using isotope ratio analysis.

Product	Tissue	Purpose *	Isotopes	Other Markers	Ref.
*Bovine*					
Zebu	Muscle *(Longissimus thoracis)*	GO, PM	*δ*^2^H, *δ*^13^C, *δ*^15^N, *δ*^18^O, *δ*^34^S	Fatty acids	[136]
Yak	Muscle (back, hip leg)	GO	*δ*^13^C, *δ*^15^N, *δ*^34^S	-	[137]
Beef	Bone	GO	*δ*^13^C, *δ*^15^N, *δ*^18^O	Na, Mg, K, V, Cr, Mn, Fe, Co, Ni, Zn, Se, Sr, Mo, Sn, Ba, Pb, Th, U	[138]
Beef	Kidney, liver	GO	^206^Pb/^204^Pb, ^207^Pb/^204^Pb, ^08^Pb/^204^Pb, ^207^Pb/^206^Pb, ^208^Pb/^206^Pb	-	
Beef	Muscle	GO	*δ*^13^C, *δ*^15^N	Be, Na, Mg, K, Ca, Ti, V, Mn, Fe, Co, Ni, Cu, Zn, Ga, Se, Rb, Sr, Zr, Mo, Sn, Sb, Ba, Bi	[139]
Beef—processed	Muscle	FR	*δ*^13^C, *δ*^15^N	-	[140]
Beef	Muscle (rib)	FR	*δ*^13^C, ^14^C	-	[141]
Beef	Muscle (patties)	GO	*δ*^13^C, *δ*^15^N	-	[56]
Beef	Muscle	GO, FR	*δ*^2^H, *δ*^13^C, *δ*^15^N, *δ*^18^O, *δ*^34^S	-	[61]
Beef	Muscle	FR	*δ*^2^H, *δ*^13^C, *δ*^15^N, *δ*^18^O, *δ*^34^S		[66]
Beef	Muscle (neck)	GO	*δ*^13^C, *δ*^15^N, *δ*^18^O, *δ*^34^S, ^87^Sr/^86^Sr	Li, B, Na, Mg, Al, K, As, Fe, Ca, V, Mn, Co, Ni, Cu, Zn, Ga, Se, Rb, Sr, Mo, Cd, Cs, Ba, La, Ce, Nd, Sm, Eu, Yb, Lu, Tl, Pb, U	[142]
Beef	Muscle (rib, sirloin)	GO	*δ*^13^C, *δ*^15^N, *δ*^18^O		[143]
Beef	Muscle	GO	*δ*^2^H, *δ*^13^C, *δ*^15^N	-	[144]
Beef—processed (roasted)	Muscle water, minced muscle water	GO	*δ*^2^H, *δ*^18^O	-	[145]
*Ovine and caprine*					
Lamb	Muscle	GO	*δ*^2^H, *δ*^13^C, *δ*^15^N, *δ*^18^O,	Sr, Y, Mo, La, Ce, Nd, Pb, Na, Mg, Al, K, Zn, Rb, fatty acids	[146]
Lamb	Muscle	GO	*δ*^13^C, *δ*^15^N	Mn, Fe, Cu, Zn, As, Rb, Sr, Mo, Cs	[147]
Lamb	Muscle (*longissimus lumborum*)	GO	*δ*^13^C, *δ*^15^N	-	[148]
Lamb	Muscle (*longissimus lumborum*)	GO, FR	*δ*^13^C, *δ*^15^N	-	[149]
Lamb	Muscle	GO, FR	*δ*^2^H, *δ*^13^C, *δ*^15^N	-	[58]
Lamb	Muscle (*longissimus dorsi*)	GO, PM	*δ*^2^H, *δ*^13^C, *δ*^15^N, *δ*^18^O, *δ*^34^S	Fatty acids	[65]
Mutton	Kidney, liver	GO	^206^Pb/^204^Pb, ^207^Pb/^204^Pb, ^208^Pb/^204^Pb, ^207^Pb/^206^Pb, ^208^Pb/^206^Pb	-	[70]
Lamb	Muscle (*longissimus thoracis*)	FR	*δ*^15^N	-	[55]
*Poultry*					
Chicken	Muscle (breast)	GO	*δ*^2^H, *δ*^13^C, *δ*^15^N, *δ*^18^O	-	[150]
Chicken, turkey	Muscle	GO	*δ*^2^H, *δ*^13^C, *δ*^15^N, *δ*^18^O, *δ*^34^S	Li, Be, B, Na, Mg, Al, Ca, Cr, Mn, Fe, Co, Ni, Cu, Zn, Ga, As, Se, Rb, Sr, Mo, Ag, Cd, Te, Ba, Tl, Pb	[57]
Chicken	Kidney, liver	GO	^206^Pb/^204^Pb, ^207^Pb/^204^Pb, ^208^Pb/^204^Pb, ^207^Pb/^206^Pb, ^208^Pb/^206^Pb	-	[70]
Chicken	Muscle (breast)	PM, FR	*δ*^13^C, *δ*^15^N	-	[151]
Chicken	Muscle (breast, thigh, drumstick, wing)	FR	*δ*^13^C, *δ*^15^N	-	[152]
Chicken	Muscle (breast)	FR	*δ*^13^C	-	[54]
*Swine*					
Pig	Muscle water (tenderloin)	GO, FR	*δ*^2^H, *δ*^13^C, *δ*^18^O	Na, Mg, K, P, Zn, Fe, Cu, Mn, Cr, Pb, Cd	[153]
Pig	Muscle	GO, PM	*δ*^2^H, *δ*^13^C, *δ*^15^N, *δ*^18^O	K, Na, Mg, Ca, Fe, Cu, Se	[154]
Pig	Muscle	GO	^7^Li/^6^Li, ^11^B/^10^B, ^53^Cr/^52^Cr, ^52^Cr/^50^Cr, ^60^Ni/^58^Ni, ^65^Cu/^64^Cu, ^71^Ga/^69^Ga, ^88^Sr/^86^Sr, ^87^Sr/^84^Sr, ^88^Sr/^84^Sr, ^87^Sr/^86^Sr, ^86^Sr/^84^Sr, ^97^Mo/^95^Mo, ^97^Mo/^94^Mo, ^95^Mo/^94^Mo, ^109^Ag/^107^Ag, ^114^Cd/^113^Cd, ^114^Cd/^112^Cd, ^114^Cd/^111^Cd, ^114^Cd/^110^Cd, ^113^Cd/^112^Cd, ^13^Cd/^111^Cd, ^113^Cd/^110^Cd, ^112^Cd/^111^Cd, ^112^Cd/^110^Cd, ^111^Cd/^110^Cd	-	[72]
Pig—processed (dry-cured hams)	Muscle (hind)	GO, PM	^87^Sr/^86^Sr	Trace and ultra-trace elements; Rb/Sr	[68]
Pig	Muscle (sirloin)	GO	*δ*^13^C, *δ*^15^N, ^11^B/^10^B, ^53^Cr/^50^Cr, ^52^Cr/^50^Cr, ^60^Ni/^58^Ni, ^65^Cu/^63^Cu, ^66^Zn/^64^Zn, ^71^Ga/^69^Ga, ^88^Sr/^86^Sr, ^88^Sr/^84^Sr, ^87^Sr/^86^Sr, ^87^Sr/^84^Sr, ^86^Sr/^84^Sr, ^97^Mo/^95^Mo, ^97^Mo/^94^Mo, ^95^Mo/^94^Mo, ^109^Ag/^107^Ag, ^208^Pb/^207^Pb, ^208^Pb/^206^Pb, ^114^Cd/^111^Cd, ^114^Cd/^110^Cd, ^113^Cd/^112^Cd, ^113^Cd/^111^Cd, ^113^Cd/^110^Cd, ^112^Cd/^111^Cd, ^112^Cd/^110^Cd, ^111^Cd/^110^Cd	V, Mn, Co, As, Se, Rb, Cs	[71]
Pig	Muscle	GO	*δ*^2^H, *δ*^13^C, *δ*^15^N, *δ*^18^O, ^87^Sr/^86^Sr		[155]
Pig	Muscle (*longissimus dorsi*)	PM	*δ*^13^C, *δ*^15^N	-	[156]
Pig—processed (dry-cured hams)	Muscle (*biceps femoris*), subcutaneous fat	GO	*δ*^2^H, *δ*^13^C, *δ*^15^N, *δ*^18^O, *δ*^34^S	-	[67]

* GO = geographical origin; FR = feeding regime; PM = production method.

**Table A3:** Works published within 2010–2022 dealing with the authenticity verification of milk and dairy products using isotope ratio analysis.

Species	Product	Fraction	Purpose *	Isotopes	Other Markers	Ref.
*Bovine*						
Cow	Milk, cheese	Fatty acids	PM	*δ*^13^C	-	[76]
Cow	Milk	Lactose, milk water	GO	*δ*^2^H, *δ*^13^C, *δ*^18^O	-	[157]
Cow	Milk	Whole, fat, casein, whey	GO	*δ*^2^H, *δ*^13^C, *δ*^15^N, *δ*^18^O	Li, Al, Cr, Mn, Fe, Co, Ni, Cu, Zn, As, Se, Sr, Cd, Ba, Pb, Bi	[158]
Cow	Milk	Whole	GO	*δ*^13^C, *δ*^15^N, *δ*^18^O	51 elements, 35 fatty acids	[100]
Cow	Milk, infant formulae	Whole	GO	*δ*^2^H, *δ*^13^C, *δ*^15^N, *δ*^18^O, *δ*^34^S, ^87^Sr/^86^Sr	N, C, S	[93]
Cow	Milk	Whole, casein	GO	*δ*^2^H, *δ*^13^C, *δ*^15^N, *δ*^18^O, *δ*^34^S, ^87^Sr/^86^Sr	Ca, Cl, K, P, S, Br, Rb, Sr	[90]
Cow	Milk	Milk water	GO	*δ*^2^H, *δ*^18^O	-	[60]
Cow	Milk	Whole, fat, casein	PM	*δ*^13^C, *δ*^15^N, *δ*^18^O, *δ*^34^S	-	[95]
Cow	Milk	Whole, casein	GO	*δ*^13^C, *δ*^15^N	Si, Se, Li, B, Rb, Ba, P, Mn, Mo, Pb	[159]
Cow	Milk	Milk water, casein, lactose	GO, SP, SEAS	*δ*^2^H, *δ*^18^O	-	[59]
Cow	Milk	Casein	GO, SEAS	*δ*^2^H, *δ*^13^C, *δ*^15^N, *δ*^18^O	-	[160]
Cow	Milk	Fat	GO, SEAS	*δ*^13^C	Fatty acids	[98]
Cow	Milk	Whole	GO	*δ*^2^H, *δ*^13^C, *δ*^15^N, *δ*^18^O	Asp, Glu, His, Ser, Arg, Gly, Thr, Pro, Ala, Val, Met, Cys, Ile, Leu, Phe, Lys, Tyr, Na, Mg, Al, K, Ca, Sc, Ti, Mn, Fe, Zn, Se, Rb, Sr, Mo	[101]
Cow	Milk	Whole	GO	*δ*^2^H, *δ*^13^C, *δ*^15^N, *δ*^18^O	-	[86]
Cow	Milk	Casein	GO, SEAS	*δ*^2^H, *δ*^13^C, *δ*^15^N, *δ*^18^O, *δ*^34^S	P, S, Cl, K, Ca, Zn, Br, Rb, Sr	[161]
Cow	Milk	Whole	GO, PM	*δ*^13^C, *δ*^15^N, *δ*^18^O, *δ*^34^S	-	[99]
Cow	Milk	Whole	GO	*δ*^2^H, *δ*^13^C, *δ*^15^N, *δ*^18^O	-	[162]
Cow	Cheese	Casein	GO, PM	*δ*^34^S	-	[63]
Cow	Cheese	Casein	GO, SP, PM	*δ*^13^C	Co, P, As, Mn, K, Li, Mg, Ga, Ca, Na, Zn, Rb	[163]
Cow	Milk	Whole, fatty acids, amino acids	PM	*δ*^13^C, *δ*^15^N	-	[164]
Cow	Milk	Whole	GO	*δ*^2^H, *δ*^13^C, *δ*^15^N, *δ*^18^O	Ag, Al, As, B, Ba, Be, Ca, Cd, Co, Cr, Cu, Fe, Hg, K, Li, Mg, Mn, Mo, Na, Ni, Pb, Rb, Se, Sr, V, Zn	[165]
Cow	Milk	Whole	SEAS	*δ*^13^C, *δ*^15^N, *δ*^18^O	-	[166]
Cow	Milk	Whole	PM	*δ*^13^C, *δ*^15^N	Fatty acids, vitamin E	[79]
Cow	Milk, skimmed milk, chocolate milk	Whole, fat	GO	*δ*^13^C, *δ*^15^N	-	[167]
Cow	Cheese	Whole, casein, fat	GO, PM	*δ*^13^C, *δ*^15^N	-	[82]
Cow	Milk	Whole	GO	*δ*^13^C, *δ*^15^N	-	[168]
Cow	UHT milk	Casein	GO	*δ*^13^C, *δ*^15^N	-	[169]
Cow	Milk, cheese	Casein	GO	*δ*^13^C, *δ*^15^N, *δ*^18^O, *δ*^34^S	P, S, Cl, K, Ca, Zn, Br, Rb, Sr	[96]
Cow	Milk	Whole	GO, SP	*δ*^2^H, *δ*^13^C, *δ*^18^O	Al, Ag, As, Ba, Be, Bi, Ca, Cd, Co, Cr, Cs, Cu, Fe, Ga, In, K, Li, Mg, Mn, Na, Ni, Pb, Rb, Se, Sr, Tl, V, U, Zn	[170]
Cow	Milk, UHT milk	Whole	GO, PM, SEAS	*δ*^13^C, *δ*^15^N	-	[171]
Cow	Milk	Proteins, milk water	GO	*δ*^2^H, *δ*^13^C, *δ*^15^N, *δ*^18^O	-	[62]
Cow	Milk, pasteurized milk, cheese	Dry matter, fat	PM	*δ*^13^C, *δ*^15^N	-	[75]
Cow	Milk, pasteurized milk	Casein, fat	PM	*δ*^13^C, *δ*^15^N	α-linolenic acid	[102]
Cow	Skimmed milk, milk powder	Fatty acids	GO	*δ*^2^H, *δ*^13^C, *δ*^15^N	Fatty acids	[172]
Cow	Milk	Fat	GO, FR	*δ*^2^H, *δ*^13^C, *δ*^15^N, *δ*^18^O	-	[172]
Cow	Milk, cheese	Whole	GO	*δ*^2^H, *δ*^13^C, *δ*^15^N, *δ*^18^O ^87^Sr/^86^Sr	-	[77]
Cow	Cheese	Whole	GO	*δ*^2^H, *δ*^13^C, *δ*^15^N, *δ*^34^S	Li, Na, Mn, Fe, Cu, Se, Rb, Sr, Mo, Ba, Re, Bi, U	[173]
Cow	Milk	Fat	FR	*δ*^13^C	Phytanic acid	[74]
Cow	Milk	Whole	PM	*δ*^13^C, *δ*^15^N	-	[78]
Cow	Milk	Fat	PM	*δ*^13^C	Fatty acids	[81]
Cow	Milk powder	Fatty acids	GO	*δ*^2^H, *δ*^13^C	-	[174]
Cow	Milk, cheese	Casein	GO, PM	*δ*^2^H, *δ*^13^C, *δ*^15^N, *δ*^18^O	-	[87]
Cow	Pasteurized milk	Protein, fat	PM	*δ*^13^C, *δ*^15^N, *δ*^34^S	-	[80]
Cow	Cheese	Casein	GO, PM	*δ*^2^H, *δ*^13^C, *δ*^15^N, *δ*^34^S	Li, Be, B, Na, Mg, P, K, Ca, V, Mn, Fe, Co, Ni, Cu, Zn, Ga, Ge, As, Se, Rb, Sr, Y, Mo, Pd, Ag, Cd, Sn, Sb, Te, Cs, Ba, La, Ce, Pr, Nd, Sm, Eu, Gd, Dy, Ho, Er, Tm, Yb, Re, Ir, Au, Hg, Pb, Bi, U	[84]
Cow	Raw milk, pasteurized milk	Casein, fat	GO, PM	*δ*^13^C, *δ*^15^N	-	[85]
Cow	Cheese	Casein, glycerol	GO	*δ*^13^C, *δ*^15^N, *δ*^18^O, *δ*^34^S	Al, B, Ba, Ca, Cr, Cu, Fe, K, Mg, Mn, Mo, Na, Ni, Sr, Zn, Ag, Be, Cd, Ce, Co, Cs, Dy, Er, Eu, Ga, Gd, Ge, Ho, Ir, La, Li, Lu, Nb, Nd, Pb, Pr, Pt, Rb, Re, Ru, Sb, Sm, Ta, Te, Tl, Tm, U, V, Yb	[94]
Cow	Milk	Whole	GO	*δ*^2^H, *δ*^18^O	-	[175]
Buffalo	Milk, cheese	Milk, casein (cheese)	GO, PM	*δ*^13^C, *δ*^15^N, *δ*^18^O,	-	[176]
Buffalo	Milk, cheese	Whole	GO, PM	*δ*^2^H, *δ*^13^C, *δ*^15^N, *δ*^18^O, *δ*^34^S	Li, Be, B, Na, Mg, Al, P, K, Ca, Ti, V, Cr, Mn, Fe, Co, Ni, Cu, Zn, Ga, Ge, As, Se, Rb, Sr, Y, Mo, Pd, Ag, Cd, In, Sn, Sb, Te, Cs, Ba, La, Ce, Pr, Nd, Sm, Eu, Gd, Dy, Ho, Er, Tm, Yb, Re, Ir, Pt, Au, Hg, Tl, Pb, Bi, Th, U	[97]
Buffalo	Milk, cheese, cream, butter	Whole	GO	*δ*^2^H, *δ*^13^C, *δ*^15^N, *δ*^18^O	-	[177]
*Ovine and caprine*						
Sheep	Milk, cheese	Whole	GO	*δ*^13^C	-	[76]
Sheep	Milk	Milk water, casein, lactose	GO, SP, SEAS	*δ*^2^H, *δ*^18^O	-	[59]
Sheep	Milk	Casein, fat	GO	*δ*^2^H, *δ*^13^C, *δ*^15^N, *δ*^18^O	Cr, Mn, Fe, Ni, Cu, Zn, Se, Rb, Sr, Cd, Ba	[178]
Sheep	Cheese	Whole, casein, fat	GO, PM	*δ*^13^C, *δ*^15^N	-	[82]
Sheep	Cheese	Whole	GO, FR	*δ*^2^H, *δ*^13^C, *δ*^15^N, *δ*^18^O, *δ*^34^S	-	[83]
Sheep	Milk, cheese	Casein	GO, SP	*δ*^13^C, *δ*^15^N, *δ*^18^O, *δ*^34^S	P, S, Cl, K, Ca, Zn, Br, Rb, Sr	[96]
Sheep	Raw milk	Casein, fat	PM	*δ*^13^C, *δ*^15^N	α-linolenic acid	[102]
Sheep	Milk	Whole	GO, SP	*δ*^2^H, *δ*^13^C, *δ*^18^O	Al, As, Ba, Be, Bi, Ca, Cd, Co, CR, Cs, Cu, Fe, Ga, In, K, Li, Mg, Mn, Ni, Pb, Rb, Se, Na, Ag, Sr, Tl, V, U, Zn	[170]
Goat	Milk	Milk water, casein, lactose	GO, SP, SEAS	*δ*^2^H, *δ*^18^O	-	[59]
Goat	Cheese	Casein	GO, SP, PM	*δ*^13^C	Co, P, As, Mn, K, Li, Mg, Ga, Ca, Na, Zn, Rb	[163]
Goat	Milk, cheese	Casein	GO	*δ*^13^C, *δ*^15^N, *δ*^18^O, *δ*^34^S	P, S, Cl, K, Ca, Zn, Br, Rb, Sr	[96]
Goat	Raw milk	Casein, fat	PM	*δ*^13^C, *δ*^15^N	α-linolenic acid	[102]
Goat	Milk, cheese	Whole	GO	*δ*^2^H, *δ*^13^C, *δ*^15^N, *δ*^18^O, ^87^Sr/^86^Sr	-	[77]
Goat	Milk powder	-	GO, PM	*δ*^13^C, *δ*^15^N	Li, Na, Mg, K, Ca, Mn, Cu, Zn, Rb, Sr, Mo, Cs, Ba	[179]

* GO = geographical origin; FR = feeding regime; PM = production method; SEAS = seasonality; SP = species.

**Table A4:** Works published within 2010–2022 dealing with the authenticity verification of egg and egg products using isotope ratio analysis.

Product	Fraction	Purpose *	Isotopes	Other Markers	Ref.
Chicken egg	Albumen	GO, PM	*δ*^13^C, *δ*^15^N	-	[103]
Chicken egg	Albumen, yolk	FR	*δ*^13^C, *δ*^15^N	-	[104]

* GO = geographical origin; FR = feeding regime; PM = production method.

## References

[1] Zhao Y., Zhang B., Chen G., Chen A., Yang S., Ye Z. (2014). Recent Developments in Application of Stable Isotope Analysis on Agro-Product Authenticity and Traceability. Food Chem..

[2] Ye H., Yang J., Xiao G., Zhao Y., Li Z., Bai W., Zeng X., Dong H. (2023). A Comprehensive Overview of Emerging Techniques and Chemometrics for Authenticity and Traceability of Animal-Derived Food. Food Chem..

[3] Pelegrino B.O., Silva R., Guimarães J.T., Coutinho N.F., Pimentel T.C., Castro B.G., Freitas M.Q., Esmerino E.A., Sant’Ana A.S., Silva M.C. (2020). Traceability: Perception and Attitudes of Artisanal Cheese Producers in Brazil. J. Dairy Sci..

[4] Drivelos S.A., Georgiou C.A. (2012). Multi-Element and Multi-Isotope-Ratio Analysis to Determine the Geographical Origin of Foods in the European Union. TrAC Trends Anal. Chem..

[5] Fanelli V., Mascio I., Miazzi M.M., Savoia M.A., De Giovanni C., Montemurro C. (2021). Molecular Approaches to Agri-Food Traceability and Authentication: An Updated Review. Foods.

[6] Vinci G., Preti R., Tieri A., Vieri S. (2013). Authenticity and Quality of Animal Origin Food Investigated by Stable-Isotope Ratio Analysis. J. Sci. Food Agric..

[7] Camin F., Bontempo L., Perini M., Piasentier E. (2016). Stable Isotope Ratio Analysis for Assessing the Authenticity of Food of Animal Origin. Compr. Rev. Food Sci. Food Saf..

[8] Prache S., Martin B., Coppa M. (2020). Authentication of Grass-Fed Meat and Dairy Products from Cattle and Sheep. Animal.

[9] Visciano P., Schirone M. (2021). Food Frauds: Global Incidents and Misleading Situations. Trends Food. Sci. Technol..

[10] European Commission Knowledge Centre for Food Fraud and Quality Monthly Food Fraud Summary Reports (January 2023, February 2023, March 2023). https://knowledge4policy.ec.europa.eu/food-fraud-quality/monthly-food-fraud-summary-reports_en#year2023.

[11] European Parliament, Council of the European Union (2002). Regulation (EC) No 178/2002 of the European Parliament and of the Council of 28 January 2002 Laying down the General Principles and Requirements of Food Law, Establishing the European Food Safety Authority and Laying down Procedures in Matters of Food Safety. Off. J. Eur. Union.

[12] European Parliament, Council of the European Union (2017). Regulation (EU) 2017/625 of the European Parliament and of the Council of 15 March 2017 on Official Controls and Other Official Activities Performed to Ensure the Application of Food and Feed Law, Rules on Animal Health and Welfare, Plant Health and Plant Protection Products. Off. J. Eur. Union.

[13] Camin F., Boner M., Bontempo L., Fauhl-Hassek C., Kelly S.D., Riedl J., Rossmann A. (2017). Stable Isotope Techniques for Verifying the Declared Geographical Origin of Food in Legal Cases. Trends Food Sci. Technol..

[14] Danezis G.P., Tsagkaris A.S., Camin F., Brusic V., Georgiou C.A. (2016). Food Authentication: Techniques, Trends & Emerging Approaches. TrAC-Trends Anal. Chem..

[15] Katerinopoulou K., Kontogeorgos A., Salmas C.E., Patakas A., Ladavos A. (2020). Geographical Origin Authentication of Agri-Food Products: A Review. Foods.

[16] Danezis G.P., Tsagkaris A.S., Brusic V., Georgiou C.A. (2016). Food Authentication: State of the Art and Prospects. Curr. Opin. Food Sci..

[17] Ghidini S., Ianieri A., Zanardi E., Conter M., Boschetti T., Iacumin P., Bracchi P.G. (2006). Stable Isotopes Determination in Food Authentication: A Review. Ann. Fac. Medic. Vet. Parma.

[18] Varrà M.O., Ghidini S., Husáková L., Ianieri A., Zanardi E. (2021). Advances in Troubleshooting Fish and Seafood Authentication by Inorganic Elemental Composition. Foods.

[19] Borràs E., Ferré J., Boqué R., Mestres M., Aceña L., Busto O. (2015). Data Fusion Methodologies for Food and Beverage Authentication and Quality Assessment—A Review. Anal. Chim. Acta.

[20] van Leeuwen K.A., Prenzler P.D., Ryan D., Camin F. (2014). Gas Chromatography-Combustion-Isotope Ratio Mass Spectrometry for Traceability and Authenticity in Foods and Beverages. Compr. Rev. Food Sci. Food Saf..

[21] Meier-Augenstein W. (1999). Applied Gas Chromatography Coupled to Isotope Ratio Mass Spectrometry. J. Chromatogr. A.

[22] Perini M., Bontempo L. (2022). Liquid Chromatography Coupled to Isotope Ratio Mass Spectrometry (LC-IRMS): A Review. TrAC Trends Anal. Chem..

[23] Yang L. (2009). Accurate and Precise Determination of Isotopic Ratios by Mc-Icp-Ms: A Review. Mass Spectrom. Rev..

[24] Yang L., Tong S., Zhou L., Hu Z., Mester Z., Meija J. (2018). A Critical Review on Isotopic Fractionation Correction Methods for Accurate Isotope Amount Ratio Measurements by MC-ICP-MS. J. Anal. At. Spectrom..

[25] Becker J.S. (2002). State-of-the-Art and Progress in Precise and Accurate Isotope Ratio Measurements by ICP-MS and LA-ICP-MS. J. Anal. At. Spectrom..

[26] Jézéquel T., Joubert V., Giraudeau P., Remaud G.S., Akoka S. (2017). The New Face of Isotopic NMR at Natural Abundance. Magn. Reson. Chem..

[27] Wang Y., Gong Y., Zhang J., Tang Y., Shi X., Shi J. (2021). Intra- and Inter-Specific Variation in Edible Jellyfish Biomarkers and Implications for Origin Traceability and Authentication. Front. Mar. Sci..

[28] Varrà M.O., Ghidini S., Zanardi E., Badiani A., Ianieri A. (2019). Authentication of European Sea Bass According to Production Method and Geographical Origin by Light Stable Isotope Ratio and Rare Earth Elements Analyses Combined with Chemometrics. Ital. J. Food Saf..

[29] del Rio-Lavín A., Weber J., Molkentin J., Jiménez E., Artetxe-Arrate I., Pardo M.Á. (2022). Stable Isotope and Trace Element Analysis for Tracing the Geographical Origin of the Mediterranean Mussel (*Mytilus Galloprovincialis*) in Food Authentication. Food Control.

[30] Tulli F., Moreno-Rojas J.M., Messina C.M., Trocino A., Xiccato G., Muñoz-Redondo J.M., Santulli A., Tibaldi E. (2020). The Use of Stable Isotope Ratio Analysis to Trace European Sea Bass (*D. labrax*) Originating from Different Farming Systems. Animals.

[31] Xu Y., Peng K., Jiang F., Cui Y.M., Han D., Liu H., Hong H., Tian X. (2022). Geographical Discrimination of Swimming Crabs (*Portunus Trituberculatus*) Using Stable Isotope and Multi-Element Analyses. J. Food Compos. Anal..

[32] Rampazzo F., Tosi F., Tedeschi P., Gion C., Arcangeli G., Brandolini V., Giovanardi O., Maietti A., Berto D. (2020). Preliminary Multi Analytical Approach to Address Geographic Traceability at the Intraspecific Level in Scombridae Family. Isotopes Environ. Health Stud..

[33] Liu Y., Liu G., Yuan Z., Liu H., Lam P.K.S. (2018). Heavy Metals (As, Hg and V) and Stable Isotope Ratios (Δ13C and Δ15N) in Fish from Yellow River Estuary, China. Sci. Total Environ..

[34] Ortea I., Gallardo J.M. (2015). Investigation of Production Method, Geographical Origin and Species Authentication in Commercially Relevant Shrimps Using Stable Isotope Ratio and/or Multi-Element Analyses Combined with Chemometrics: An Exploratory Analysis. Food Chem..

[35] Soto D.X., Hobson K.A., Wassenaar L.I. (2016). Using Hydrogen Isotopes of Freshwater Fish Tissue as a Tracer of Provenance. Ecol. Evol..

[36] Won E.J., Kim S.H., Go Y.S., Kumar K.S., Kim M.S., Yoon S.H., Bayon G., Kim J.H., Shin K.H. (2021). A Multi-Elements Isotope Approach to Assess the Geographic Provenance of Manila Clams (*Ruditapes philippinarum*) via Recombining Appropriate Elements. Foods.

[37] Martino J.C., Trueman C.N., Mazumder D., Crawford J., Doubleday Z.A. (2022). The Universal Imprint of Oxygen Isotopes Can Track the Origins of Seafood. Fish Fish..

[38] Kang X., Zhao Y., Tan Z., Ning J., Zhai Y., Zheng G. (2022). Evaluation of Multivariate Data Analysis for Marine Mussels Mytilus Edulis Authentication in China: Based on Stable Isotope Ratio and Compositions of C, N, O and H. J. Food Compos. Anal..

[39] Brombin V., Natali C., Frijia G., Schmitt K., Casalini M., Bianchini G. (2022). Isotope Geochemistry for Seafood Traceability and Authentication: The Northern Adriatic Manila Clams Case Study. Foods.

[40] Camin F., Perini M., Bontempo L., Galeotti M., Tibaldi E., Piasentier E. (2018). Stable Isotope Ratios of H, C, O, N and S for the Geographical Traceability of Italian Rainbow Trout (*Oncorhynchus Mykiss*). Food Chem..

[41] Han C., Dong S., Li L., Gao Q. (2021). Efficacy of Using Stable Isotopes Coupled with Chemometrics to Differentiate the Production Method and Geographical Origin of Farmed Salmonids. Food Chem..

[42] Shipley O.N., Newton A.L., Frisk M.G., Henkes G.A., LaBelle J.S., Camhi M.D., Hyatt M.W., Walters H., Olin J.A. (2021). Telemetry-Validated Nitrogen Stable Isotope Clocks Identify Ocean-to-Estuarine Habitat Shifts in Mobile Organisms. Methods Ecol. Evol..

[43] Phillips D.L., Eldridge P.M. (2006). Estimating the Timing of Diet Shifts Using Stable Isotopes. Oecologia.

[44] Busst G.M.A., Britton J.R. (2018). Tissue-Specific Turnover Rates of the Nitrogen Stable Isotope as Functions of Time and Growth in a Cyprinid Fish. Hydrobiologia.

[45] Chen H.L., Chang N.N., Hsiao W.V., Chen W.J., Wang C.H., Shiao J.C. (2022). Using Molecular Phylogenetic and Stable Isotopic Analysis to Identify Species, Geographical Origin and Production Method of Mullet Roes. Food Control.

[46] Thomatou A.A., Psarra E., Mazarakioti E.C., Katerinopoulou K., Tsirogiannis G., Zotos A., Kontogeorgos A., Patakas A., Ladavos A. (2022). Stable Isotope Analysis for the Discrimination of the Geographical Origin of Greek Bottarga ‘Avgotaracho Messolongiou’: A Preliminary Research. Foods.

[47] Zhao L., Tanaka K., Tazoe H., Iizuka T., Kubota K., Murakami-Sugihara N., Shirai K. (2019). Determination of the Geographical Origin of Marine Mussels (*Mytilus* Spp.) Using ^143^Nd/^144^Nd Ratios. Mar. Environ. Res..

[48] Tanaka K., Zhao L., Tazoe H., Iizuka T., Murakami-Sugihara N., Toyama K., Yamamoto T., Yorisue T., Shirai K. (2022). Using Neodymium Isotope Ratio in *Ruditapes philippinarum* Shells for Tracking the Geographical Origin. Food Chem..

[49] Zhang X., Cheng J., Han D., Zhao X., Chen X., Liu Y. (2019). Geographical Origin Traceability and Species Identification of Three Scallops (Patinopecten Yessoensis, Chlamys Farreri, and Argopecten Irradians) Using Stable Isotope Analysis. Food Chem..

[50] Zhao X., Liu Y., Wang G., Tao W., Lou Y., Li N., Liu Y. (2019). Tracing the Geographical Origins of Yesso Scallop (Patinopecten Yessoensis) by Using Compound-Specific Isotope Analysis: An Approach for Overcoming the Seasonal Effect. Food Control.

[51] Go Y.S., Won E.J., Kim S.H., Lee D.H., Kang J.H., Shin K.H. (2022). Stepwise Approach for Tracing the Geographical Origins of the Manila Clam *Ruditapes philippinarum* Using Dual-Element Isotopes and Carbon Isotopes of Fatty Acids. Foods.

[52] Dang C., De Montaudouin X., Savoye N., Caill-Milly N., Martinez P., Sauriau P.G. (2009). Stable Isotopes Changes in the Adductor Muscle of Diseased Bivalve *Ruditapes philippinarum*. Mar. Biol..

[53] Pianezze S., Camin F., Perini M., Corazzin M., Piasentier E. (2021). Tracing Lamb Meat with Stable Isotope Ratio Analysis: A Review. Small Rumin. Res..

[54] Rhodes C.N., Lofthouse J.H., Hird S., Rose P., Reece P., Christy J., Macarthur R., Brereton P.A. (2010). The Use of Stable Carbon Isotopes to Authenticate Claims That Poultry Have Been Corn-Fed. Food Chem..

[55] Devincenzi T., Delfosse O., Andueza D., Nabinger C., Prache S. (2014). Dose-Dependent Response of Nitrogen Stable Isotope Ratio to Proportion of Legumes in Diet to Authenticate Lamb Meat Produced from Legume-Rich Diets. Food Chem..

[56] Martinelli L.A., Nardoto G.B., Chesson L.A., Rinaldi F.D., Ometto J.P.H.B., Cerling T.E., Ehleringer J.R. (2011). Worldwide Stable Carbon and Nitrogen Isotopes of Big Mac^®^ Patties: An Example of a Truly “Glocal” Food. Food Chem..

[57] Rees G., Kelly S.D., Cairns P., Ueckermann H., Hoelzl S., Rossmann A., Scotter M.J. (2016). Verifying the Geographical Origin of Poultry: The Application of Stable Isotope and Trace Element (SITE) Analysis. Food Control.

[58] Sun S., Guo B., Wei Y. (2016). Origin Assignment by Multi-Element Stable Isotopes of Lamb Tissues. Food Chem..

[59] Gregorčič S.H., Potočnik D., Camin F., Ogrinc N. (2020). Milk Authentication: Stable Isotope Composition of Hydrogen and Oxygen in Milks and Their Constituents. Molecules.

[60] Boito M., Iacumin P., Rossi M., Ogrinc N., Venturelli G. (2021). Isotope Partitioning between Cow Milk and Farm Water: A Tool for Verification of Milk Provenance. Rapid Commun. Mass Spectrom..

[61] Osorio M.T., Moloney A.P., Schmidt O., Monahan F.J. (2011). Beef Authentication and Retrospective Dietary Verification Using Stable Isotope Ratio Analysis of Bovine Muscle and Tail Hair. J. Agric. Food Chem..

[62] Luo D., Dong H., Luo H., Xian Y., Guo X., Wu Y. (2016). Multi-Element (C, N, H, O) Stable Isotope Ratio Analysis for Determining the Geographical Origin of Pure Milk from Different Regions. Food Anal. Methods.

[63] Pianezze S., Bontempo L., Perini M., Tonon A., Ziller L., Franceschi P., Camin F. (2020). δ^34^S for Tracing the Origin of Cheese and Detecting Its Authenticity. J. Mass Spectrom..

[64] Zazzo A., Monahan F.J., Moloney A.P., Green S., Schmidt O. (2011). Sulphur Isotopes in Animal Hair Track Distance to Sea. Rapid Commun. Mass Spectrom..

[65] Mekki I., Camin F., Perini M., Smeti S., Hajji H., Mahouachi M., Piasentier E., Atti N. (2016). Differentiating the Geographical Origin of Tunisian Indigenous Lamb Using Stable Isotope Ratio and Fatty Acid Content. J. Food Compos. Anal..

[66] Osorio M.T., Moloney A.P., Schmidt O., Monahan F.J. (2011). Multielement Isotope Analysis of Bovine Muscle for Determination of International Geographical Origin of Meat. J. Agric. Food Chem..

[67] Perini M., Camin F., Sánchez Del Pulgar J., Piasentier E. (2013). Effect of Origin, Breeding and Processing Conditions on the Isotope Ratios of Bioelements in Dry-Cured Ham. Food Chem..

[68] Epova E.N., Bérail S., Zuliani T., Malherbe J., Sarthou L., Valiente M., Donard O.F.X. (2018). ^87^Sr/^86^Sr Isotope Ratio and Multielemental Signatures as Indicators of Origin of European Cured Hams: The Role of Salt. Food Chem..

[69] Coelho I., Castanheira I., Bordado J.M., Donard O., Silva J.A.L. (2017). Recent Developments and Trends in the Application of Strontium and Its Isotopes in Biological Related Fields. TrAC Trends Anal. Chem..

[70] Evans J.A., Pashley V., Richards G.J., Brereton N., Knowles T.G. (2015). Geogenic Lead Isotope Signatures from Meat Products in Great Britain: Potential for Use in Food Authentication and Supply Chain Traceability. Sci. Total Environ..

[71] Park Y.M., Lee C.M., Hong J.H., Jamila N., Khan N., Jung J.H., Jung Y.C., Kim K.S. (2018). Origin Discrimination of Defatted Pork via Trace Elements Profiling, Stable Isotope Ratios Analysis, and Multivariate Statistical Techniques. Meat Sci..

[72] Nho E.Y., Choi J.Y., Lee C.M., Dang Y.M., Khan N., Jamila N., Kim K.S. (2019). Origin Authentication of Pork Fat via Elemental Composition, Isotope Ratios, and Multivariate Chemometric Analyses. Anal. Lett..

[73] Besser A.C., Elliott Smith E.A., Newsome S.D. (2022). Assessing the Potential of Amino Acid δ^13^C and δ^15^N Analysis in Terrestrial and Freshwater Ecosystems. J. Ecol..

[74] Kaffarnik S., Schröder M., Lehnert K., Baars T., Vetter W. (2014). Δ13C Values and Phytanic Acid Diastereomer Ratios: Combined Evaluation of Two Markers Suggested for Authentication of Organic Milk and Dairy Products. Eur. Food Res. Technol..

[75] Capici C., Mimmo T., Kerschbaumer L., Cesco S., Scampicchio M. (2015). Determination of Cheese Authenticity by Carbon and Nitrogen Isotope Analysis: Stelvio Cheese as a Case Study. Food Anal. Methods.

[76] Schipilliti L., Bonaccorsi I., Consolo G., Mondello L. (2022). Isotopic and Statistical Methods for the Traceability of Milk and Dairy Products. Food Anal. Methods.

[77] Stevenson R., Desrochers S., Hélie J.F. (2015). Stable and Radiogenic Isotopes as Indicators of Agri-Food Provenance: Insights from Artisanal Cheeses from Quebec, Canada. Int. Dairy J..

[78] Chung I.M., Park I., Yoon J.Y., Yang Y.S., Kim S.H. (2014). Determination of Organic Milk Authenticity Using Carbon and Nitrogen Natural Isotopes. Food Chem..

[79] Chung I.M., Kim J.K., Lee K.J., Son N.Y., An M.J., Lee J.H., An Y.J., Kim S.H. (2018). Discrimination of Organic Milk by Stable Isotope Ratio, Vitamin E, and Fatty Acid Profiling Combined with Multivariate Analysis: A Case Study of Monthly and Seasonal Variation in Korea for 2016–2017. Food Chem..

[80] Molkentin J., Giesemann A. (2010). Follow-up of Stable Isotope Analysis of Organic versus Conventional Milk. Anal. Bionanal. Chem..

[81] Molkentin J. (2013). Applicability of Organic Milk Indicators to the Authentication of Processed Products. Food Chem..

[82] Faberi A., Compagnone D., Fuselli F., La Mantia A., Mascini M., Montesano C., Rocchi R., Sergi M. (2018). Italian Cheeses Discrimination by Means of δ^13^C and δ^15^N Isotopic Ratio Mass Spectrometry. Food Anal. Methods.

[83] Valenti B., Biondi L., Campidonico L., Bontempo L., Luciano G., Di Paola F., Copani V., Ziller L., Camin F. (2017). Changes in Stable Isotope Ratios in PDO Cheese Related to the Area of Production and Green Forage Availability. The Case Study of Pecorino Siciliano. Rapid Commun. Mass Spectrom..

[84] Camin F., Wehrens R., Bertoldi D., Bontempo L., Ziller L., Perini M., Nicolini G., Nocetti M., Larcher R. (2012). H, C, N and S Stable Isotopes and Mineral Profiles to Objectively Guarantee the Authenticity of Grated Hard Cheeses. Anal. Chim. Acta.

[85] Scampicchio M., Mimmo T., Capici C., Huck C., Innocente N., Drusch S., Cesco S. (2012). Identification of Milk Origin and Process-Induced Changes in Milk by Stable Isotope Ratio Mass Spectrometry. J. Agric. Food Chem..

[86] Behkami S., Gholami R., Gholami M., Roohparvar R. (2020). Precipitation Isotopic Information: A Tool for Building the Data Base to Verify Milk Geographical Origin Traceability. Food Control.

[87] Bontempo L., Lombardi G., Paoletti R., Ziller L., Camin F. (2012). H, C, N and O Stable Isotope Characteristics of Alpine Forage, Milk and Cheese. Int. Dairy J..

[88] Vander Zanden H.B., Soto D.X., Bowen G.J., Hobson K.A. (2016). Expanding the Isotopic Toolbox: Applications of Hydrogen and Oxygen Stable Isotope Ratios to Food Web Studies. Front. Ecol. Evol..

[89] Krivachy Tanz N., Rossmann A., Schmidt H.L. (2015). Potentials and Caveats with Oxygen and Sulfur Stable Isotope Analyses in Authenticity and Origin Checks of Food and Food Commodities. Food Control.

[90] Gregorčič S.H., Ogrinc N., Frew R., Nečemer M., Strojnik L., Zuliani T. (2021). The Provenance of Slovenian Milk Using ^87^sr/^86^sr Isotope Ratios. Foods.

[91] Baffi C., Trincherini P.R. (2016). Food Traceability Using the ^87^Sr/^86^Sr Isotopic Ratio Mass Spectrometry. Eur. Food Res. Technol..

[92] Flockhart D.T.T., Kyser T.K., Chipley D., Miller N.G., Norris D.R. (2015). Experimental Evidence Shows No Fractionation of Strontium Isotopes (^87^Sr/^86^Sr) among Soil, Plants, and Herbivores: Implications for Tracking Wildlife and Forensic Science. Isotopes Environ. Health Stud..

[93] Zhou X., Yan Z., Jin B., Wu Y., Xie L., Chen H., Lin G., Zhao Y., Rogers K.M., Wu H. (2021). Origin Verification of Imported Infant Formula and Fresh Milk into China Using Stable Isotope and Elemental Chemometrics. Food Control.

[94] Bontempo L., Larcher R., Camin F., Hölzl S., Rossmann A., Horn P., Nicolini G. (2011). Elemental and Isotopic Characterisation of Typical Italian Alpine Cheeses. Int. Dairy J..

[95] O’Sullivan R., Monahan F.J., Bahar B., Kirwan L., Pierce K., O’Shea A., Mc Elroy S., Malone F., Hanafin B., Molloy S. (2021). Stable Isotope Profile (C, N, O, S) of Irish Raw Milk: Baseline Data for Authentication. Food Control.

[96] Nečemer M., Potočnik D., Ogrinc N. (2016). Discrimination between Slovenian Cow, Goat and Sheep Milk and Cheese According to Geographical Origin Using a Combination of Elemental Content and Stable Isotope Data. J. Food Compos. Anal..

[97] Bontempo L., Barbero A., Bertoldi D., Camin F., Larcher R., Perini M., Sepulcri A., Zicarelli L., Piasentier E. (2019). Isotopic and Elemental Profiles of Mediterranean Buffalo Milk and Cheese and Authentication of Mozzarella Di Bufala Campana PDO: An Initial Exploratory Study. Food Chem..

[98] Potočnik D., Strojnik L., Eftimov T., Levart A., Ogrinc N. (2020). Fatty Acid and Stable Carbon Isotope Composition of Slovenian Milk: Year, Season, and Regional Variability. Molecules.

[99] Chung I.M., Kim J.K., Yang Y.J., An Y.J., Kim S.Y., Kwon C., Kim S.H. (2020). A Case Study for Geographical Indication of Organic Milk in Korea Using Stable Isotope Ratios-Based Chemometric Analysis. Food Control.

[100] Xu S., Zhao C., Deng X., Zhang R., Qu L., Wang M., Ren S., Wu H., Yue Z., Niu B. (2021). Determining the Geographical Origin of Milk by Multivariate Analysis Based on Stable Isotope Ratios, Elements and Fatty Acids. Anal. Methods.

[101] Xie L., Zhao S., Rogers K.M., Xia Y., Zhang B., Suo R., Zhao Y. (2020). A Case of Milk Traceability in Small-Scale Districts-Inner Mongolia of China by Nutritional and Geographical Parameters. Food Chem..

[102] Erich S., Schill S., Annweiler E., Waiblinger H.U., Kuballa T., Lachenmeier D.W., Monakhova Y.B. (2015). Combined Chemometric Analysis of 1H NMR, 13C NMR and Stable Isotope Data to Differentiate Organic and Conventional Milk. Food Chem..

[103] Rogers K.M., Van Ruth S., Alewijn M., Philips A., Rogers P. (2015). Verification of Egg Farming Systems from the Netherlands and New Zealand Using Stable Isotopes. J. Agric. Food Chem..

[104] Sun F.M., Shi G.Y., Wang H.W. (2016). Differentiation of Pigment in Eggs Using Carbon (^13^C/^12^C) and Nitrogen (^15^N/^14^N) Stable Isotopes. J. AOAC Int..

[105] Donarski J., Camin F., Fauhl-Hassek C., Posey R., Sudnik M. (2019). Sampling Guidelines for Building and Curating Food Authenticity Databases. Trends Food Sci. Technol..

[106] Xia Y., Jia L., Zhang K., Xie J., Yu E., Tian J., Gong W., Li Z., Li H., Wang G. (2022). Geographical Origin Traceability of Procambarus Clarkii Based on Mineral Elements and Stable Isotopes. Foods.

[107] Li L., Kokkuar N., Han C., Ren W., Dong S. (2020). Effects of Dietary Shifts on the Stable Isotope Signature of Pacific White Shrimp Litopenaeus Vannamei and Implications for Traceability. Mar. Freshw. Res..

[108] Gopi K., Mazumder D., Sammut J., Saintilan N., Crawford J., Gadd P. (2019). Combined Use of Stable Isotope Analysis and Elemental Profiling to Determine Provenance of Black Tiger Prawns (Penaeus Monodon). Food Control.

[109] Luo R., Jiang T., Chen X., Zheng C., Liu H., Yang J. (2019). Determination of Geographic Origin of Chinese Mitten Crab (Eriocheir Sinensis) Using Integrated Stable Isotope and Multi-Element Analyses. Food Chem..

[110] Li L., Ren W., Dong S., Feng J. (2018). Investigation of Geographic Origin, Salinity and Feed on Stable Isotope Profile of Pacific White Shrimp (Litopenaeus Vannamei). Aquac. Res..

[111] Kim H., Kumar K.S., Hwang S.Y., Kang B.C., Moon H.B., Shin K.H. (2015). Utility of Stable Isotope and Cytochrome Oxidase I Gene Sequencing Analyses in Inferring Origin and Authentication of Hairtail Fish and Shrimp. J. Agric. Food Chem..

[112] Carter J.F., Tinggi U., Yang X., Fry B. (2015). Stable Isotope and Trace Metal Compositions of Australian Prawns as a Guide to Authenticity and Wholesomeness. Food Chem..

[113] Dong X., Han C., Li L. (2022). Stable Isotope Ratio Analysis for the Authentication of Sea Urchin (Mesocentrotus Nudus) from Different Culture Areas in the North Yellow Sea, China. Aquaculture.

[114] Kang X., Zhao Y., Liu W., Ding H., Zhai Y., Ning J., Sheng X. (2021). Geographical Traceability of Sea Cucumbers in China via Chemometric Analysis of Stable Isotopes and Multi-Elements. J. Food Compos. Anal..

[115] Zhao X., Liu Y., Li Y., Zhang X., Qi H. (2018). Authentication of the Sea Cucumber (*Apostichopus Japonicus*) Using Amino Acids Carbon Stable Isotope Fingerprinting. Food Control.

[116] Zhang X., Liu Y., Li Y., Zhao X. (2017). Identification of the Geographical Origins of Sea Cucumber (*Apostichopus Japonicus*) in Northern China by Using Stable Isotope Ratios and Fatty Acid Profiles. Food Chem..

[117] Ni X., Li X., Ran G., Chen J., Jiang X., Sun J., Bai W. (2022). Determination of the Geographical Origin of *Trachinotus Ovatus* and *Pampus Argenteus* in China by Multi-Element and Stable Isotope Analysis. Food Chem..

[118] Liu Z., Yuan Y., Zhao Y., Zhang Y., Nie J., Shao S., Rogers K.M. (2020). Differentiating Wild, Lake-Farmed and Pond-Farmed Carp Using Stable Isotope and Multi-Element Analysis of Fish Scales with Chemometrics. Food Chem..

[119] Vasconi M., Lopez A., Galimberti C., Moreno Rojas J.M., Muñoz Redondo J.M., Bellagamba F., Moretti V.M. (2019). Authentication of Farmed and Wild European Eel (*Anguilla Anguilla*) by Fatty Acid Profile and Carbon and Nitrogen Isotopic Analyses. Food Control.

[120] Wang Y.V., Wan A.H.L., Lock E.J., Andersen N., Winter-Schuh C., Larsen T. (2018). Know Your Fish: A Novel Compound-Specific Isotope Approach for Tracing Wild and Farmed Salmon. Food Chem..

[121] Farabegoli F., Pirini M., Rotolo M., Silvi M., Testi S., Ghidini S., Zanardi E., Remondini D., Bonaldo A., Parma L. (2018). Toward the Authentication of European Sea Bass Origin through a Combination of Biometric Measurements and Multiple Analytical Techniques. J. Agric. Food Chem..

[122] Carrera M., Gallardo J.M. (2017). Determination of the Geographical Origin of All Commercial Hake Species by Stable Isotope Ratio (SIR) Analysis. J. Agric. Food Chem..

[123] Coulter D.P., Bowen G.J., Höök T.O. (2017). Influence of Diet and Ambient Water on Hydrogen and Oxygen Stable Isotope Ratios in Fish Tissue: Patterns within and among Tissues and Relationships with Growth Rates. Hydrobiologia.

[124] Chaguri M.P., Maulvault A.L., Costa S., Gonçalves A., Nunes M.L., Carvalho M.L., Sant’Ana L.S., Bandarra N., Marques A. (2017). Chemometrics Tools to Distinguish Wild and Farmed Meagre (*Argyrosomus Regius*). J. Food Process. Preserv..

[125] Cambiè G., Kaiser M.J., Marriott A.L., Fox J., Lambert G., Hiddink J.G., Overy T., Bennet S.A., Leng M.J., Mc Carthy I.D. (2016). Stable Isotope Signatures Reveal Small-Scale Spatial Separation in Populations of European Sea Bass. Mar. Ecol. Prog. Ser..

[126] Molkentin J., Lehmann I., Ostermeyer U., Rehbein H. (2015). Traceability of Organic Fish—Authenticating the Production Origin of Salmonids by Chemical and Isotopic Analyses. Food Control.

[127] Chaguri M.P., Maulvault A.L., Nunes M.L., Santiago D.A., Denadai J.C., Fogaça F.H., Sant’Ana L.S., Ducatti C., Bandarra N., Carvalho M.L. (2015). Different Tools to Trace Geographic Origin and Seasonality of Croaker *(Micropogonias Furnieri*). LWT-Food Sci. Technol..

[128] Kim H., Suresh Kumar K., Shin K.H. (2015). Applicability of Stable C and N Isotope Analysis in Inferring the Geographical Origin and Authentication of Commercial Fish (Mackerel, Yellow Croaker and Pollock). Food Chem..

[129] Monteiro Oliveira E.J.V., Sant’Ana L.S., Ducatti C., Denadai J.C., de Souza Kruliski C.R. (2011). The Use of Stable Isotopes for Authentication of Gadoid Fish Species. Europ.Food Res. Technol..

[130] Martín-Pérez M., Fernández-Borràs J., Ibarz A., Felip O., Gutiérrez J., Blasco J. (2011). Stable Isotope Analysis Combined with Metabolic Indices Discriminates between Gilthead Sea Bream (*Sparus Aurata*) Fingerlings Produced in Various Hatcheries. J. Agric. Food Chem..

[131] Sant’Ana L.S., Ducatti C., Ramires D.G. (2010). Seasonal Variations in Chemical Composition and Stable Isotopes of Farmed and Wild Brazilian Freshwater Fish. Food Chem..

[132] Bianchini G., Brombin V., Carlino P., Mistri E., Natali C., Salani G.M. (2021). Traceability and Authentication of Manila Clams from North-Western Adriatic Lagoons Using C and N Stable Isotope Analysis. Molecules.

[133] Zhang X., Han D., Chen X., Zhao X., Cheng J., Liu Y. (2019). Combined Use of Fatty Acid Profile and Fatty Acid δ^13^C Fingerprinting for Origin Traceability of Scallops (*Patinopecten Yessoensis*, *Chlamys Farreri*, and *Argopecten Irradians*). Food Chem..

[134] Martino J.C., Mazumder D., Gadd P., Doubleday Z.A. (2022). Tracking the Provenance of Octopus Using Isotopic and Multi-Elemental Analysis. Food Chem..

[135] Gong Y., Li Y., Chen X., Chen L. (2018). Potential Use of Stable Isotope and Fatty Acid Analyses for Traceability of Geographic Origins of Jumbo Squid (*Dosidicus Gigas*). Rapid Commun. Mass Spectrom..

[136] Perini M., Nfor M.B., Camin F., Pianezze S., Piasentier E. (2021). Using Bioelements Isotope Ratios and Fatty Acid Composition to Deduce Beef Origin and Zebu Feeding Regime in Cameroon. Molecules.

[137] Nie J., Shao S., Xia W., Liu Z., Yu C., Li R., Wang W., Li J., Yuan Y., Rogers K.M. (2020). Stable Isotopes Verify Geographical Origin of Yak Meat from Qinghai-Tibet Plateau. Meat Sci..

[138] Jiang D., Du L., Guo Y., Ma J., Li X., Han L., Xu Y., Qian Y. (2020). Potential Use of Stable Isotope and Multi-Element Analyses for Regional Geographical Traceability of Bone Raw Materials for Gelatin Production. Food Anal. Methods.

[139] Zhao Y., Zhang B., Chen G., Chen A., Yang S., Ye Z. (2013). Tracing the Geographic Origin of Beef in China on the Basis of the Combination of Stable Isotopes and Multielement Analysis. J. Agric. Food Chem..

[140] Yanagi Y., Hirooka H., Oishi K., Choumei Y., Hata H., Arai M., Kitagawa M., Gotoh T., Inada S., Kumagai H. (2012). Stable Carbon and Nitrogen Isotope Analysis as a Tool for Inferring Beef Cattle Feeding Systems in Japan. Food Chem..

[141] Kim S.H., Cruz G.D., Fadel J.G., Clifford A.J. (2012). Food Authenticity Using Natural Carbon Isotopes (12C, 13C, 14C) in Grass-Fed and Grain-Fed Beef. Food Sci. Biotechnol..

[142] Baroni M.V., Podio N.S., Badini R.G., Inga M., Ostera H.A., Cagnoni M., Gallegos E., Gautier E., Peral-García P., Hoogewerff J. (2011). How Much Do Soil and Water Contribute to the Composition of Meat? A Case Study: Meat from Three Areas of Argentina. J. Agric. Food Chem..

[143] Bong Y.S., Shin W.J., Lee A.R., Kim Y.S., Kim K., Lee K.S. (2010). Tracing the Geographical Origin of Beefs Being Circulated in Korean Markets Based on Stable Isotopes. Rapid Commun. Mass Spectrom..

[144] Horacek M., Min J.S. (2010). Discrimination of Korean Beef from Beef of Other Origin by Stable Isotope Measurements. Food Chem..

[145] Horacek M., Eisinger E., Papesch W. (2010). Reliability of Stable Isotope Values from Meat Juice for the Determination of the Meat Origin. Food Chem..

[146] Qie M., Zhang B., Li Z., Zhao S., Zhao Y. (2021). Data Fusion by Ratio Modulation of Stable Isotope, Multi-Element, and Fatty Acids to Improve Geographical Traceability of Lamb. Food Control.

[147] Liu H., Qin Y., Ma Q., Zhao Q., Guo X., Ma L., Gou C., Xia Y., Gan R.Y., Zhang J. (2021). Discrimination the Geographical Origin of Yanchi Tan Lamb with Different Muscle Sections by Stable Isotopic Ratios and Elemental Profiles. Int. J. Food Sci. Technol..

[148] Erasmus S.W., Muller M., Butler M., Hoffman L.C. (2018). The Truth Is in the Isotopes: Authenticating Regionally Unique South African Lamb. Food Chem..

[149] Erasmus S.W., Muller M., Van Der Rijst M., Hoffman L.C. (2016). Stable Isotope Ratio Analysis: A Potential Analytical Tool for the Authentication of South African Lamb Meat. Food Chem..

[150] Zhaxi C., Zhao S., Zhang T., Dong H., Liu H., Zhao Y. (2021). Stable Isotopes Verify Geographical Origin of Tibetan Chicken. Food Chem..

[151] Coletta L.D., Pereira A.L., Coelho A.A.D., Savino V.J.M., Menten J.F.M., Correr E., Frana L.C., Martinelli L.A. (2012). Barn vs. Free-Range Chickens: Differences in Their Diets Determined by Stable Isotopes. Food Chem..

[152] Cruz V.C., Araújo P.C., Sartori J.R., Pezzato A.C., Denadai J.C., Polycarpo G.V., Zanetti L.H., Ducatti C. (2012). Poultry Offal Meal in Chicken: Traceability Using the Technique of Carbon (13C12C)- and Nitrogen (15N/14N)-Stable Isotopes. Poult. Sci..

[153] Cristea G., Voica C., Feher I., Puscas R., Magdas D.A. (2022). Isotopic and Elemental Characterization of Romanian Pork Meat in Corroboration with Advanced Chemometric Methods: A First Exploratory Study. Meat Sci..

[154] Zhao Y., Tu T., Tang X., Zhao S., Qie M., Chen A., Yang S. (2020). Authentication of Organic Pork and Identification of Geographical Origins of Pork in Four Regions of China by Combined Analysis of Stable Isotopes and Multi-Elements. Meat Sci..

[155] Shin W.J., Choi S.H., Ryu J.S., Song B.Y., Song J.H., Park S., Min J.S. (2018). Discrimination of the Geographic Origin of Pork Using Multi-Isotopes and Statistical Analysis. Rapid Commun. Mass Spectrom..

[156] Zhao Y., Yang S., Wang D. (2016). Stable Carbon and Nitrogen Isotopes as a Potential Tool to Differentiate Pork from Organic and Conventional Systems. J. Sci. Food Agric..

[157] Perini M., Thomas F., Cabañero Ortiz A.I., Simoni M., Camin F. (2022). Stable Isotope Ratio Analysis of Lactose as a Possible Potential Geographical Tracer of Milk. Food Control.

[158] Kalpage M., Dissanayake C., Diyabalanage S., Chandrajith R., Frew R., Fernando R. (2022). Stable Isotope and Element Profiling for Determining the Agroclimatic Origin of Cow Milk within a Tropical Country. Foods.

[159] Ng W.L., Bay L.J., Goh G., Ang T.H., Kong K., Chew P., Koh S.P., Ch’ng A.L., Phang H., Chiew P. (2021). Multivariate Statistical Analysis of Stable Isotope Signatures and Element Concentrations to Differentiate the Geographical Origin of Retail Milk Sold in Singapore. Food Control.

[160] Wijenayake K., Frew R., Mc Comb K., Van Hale R., Clarke D. (2020). Feasibility of Casein to Record Stable Isotopic Variation of Cow Milk in New Zealand. Molecules.

[161] Potočnik D., Nečemer M., Perišić I., Jagodic M., Mazej D. (2020). Geographical Verification of Slovenian Milk Using Stable Isotope Ratio, Multi- Element and Multivariate Modelling Approaches. Food Chem..

[162] Zhao S., Zhao Y., Rogers K.M., Chen G., Chen A., Yang S. (2020). Application of Multi-Element (C, N, H, O) Stable Isotope Ratio Analysis for the Traceability of Milk Samples from China. Food Chem..

[163] Magdas D.A., Feher I., Cristea G., Voica C., Tabaran A., Mihaiu M., Cordea D.V., Bâlteanu V.A., Dan S.D. (2019). Geographical Origin and Species Differentiation of Transylvanian Cheese. Comparative Study of Isotopic and Elemental Profiling vs. DNA Results Food Chem..

[164] Chung I.M., Kim J.K., Yarnes C.T., An Y.J., Kwon C., Kim S.Y., Yang Y.J., Chi H.Y., Kim S.H. (2019). Fatty Acid- and Amino Acid-Specific Isotope Analysis for Accurate Authentication and Traceability in Organic Milk. J. Agric. Food Chem..

[165] Griboff J., Baroni M.V., Horacek M., Wunderlin D.A., Monferran M.V. (2019). Multielemental + isotopic Fingerprint Enables Linking Soil, Water, Forage and Milk Composition, Assessing the Geographical Origin of Argentinean Milk. Food Chem..

[166] Garbaras A., Skipitytė R., Šapolaitė J., Ežerinskis Ž., Remeikis V. (2019). Seasonal Variation in Stable Isotope Ratios of Cow Milk in Vilnius Region, Lithuania. Animals.

[167] Bostic J.N., Hagopian W.M., Jahren A.H. (2018). Carbon and Nitrogen Stable Isotopes in U.S. Milk: Insight into Production Process. Rapid Commun. Mass Spectrom..

[168] Behkami S., Zain S.M., Gholami M., Bakirdere S. (2017). Isotopic Ratio Analysis of Cattle Tail Hair: A Potential Tool in Building the Database for Cow Milk Geographical Traceability. Food Chem..

[169] Dong H., Xiao K., Luo D. (2017). Stability of Carbon and Nitrogen Isotopic Compositions of the Protein Extracted from Milk and Their Potential as “Fingerprints” of Geographical Origin. RSC Adv..

[170] Magdas D.A., Dehelean A., Feher I., Cristea G., Puscas R., Dan S.D., Cordea D.V. (2016). Discrimination Markers for the Geographical and Species Origin of Raw Milk within Romania. Int. Dairy J..

[171] Scampicchio M., Eisenstecken D., De Benedictis L., Capici C., Ballabio D., Mimmo T., Robatscher P., Kerschbaumer L., Oberhuber M., Kaser A. (2016). Multi-Method Approach to Trace the Geographical Origin of Alpine Milk: A Case Study of Tyrol Region. Food Anal. Methods.

[172] Ehtesham E., Hayman A., Van Hale R., Frew R. (2015). Influence of Feed and Water on the Stable Isotopic Composition of Dairy Milk. Int. Dairy J..

[173] Camin F., Bertoldi D., Santato A., Bontempo L., Perini M., Ziller L., Stroppa A., Larcher R. (2015). Validation of Methods for H, C, N and S Stable Isotopes and Elemental Analysis of Cheese: Results of an International Collaborative Study. Rapid Commun. Mass Spectrom..

[174] Ehtesham E., Hayman A.R., Mc Comb K.A., Van Hale R., Frew R.D. (2013). Correlation of Geographical Location with Stable Isotope Values of Hydrogen and Carbon of Fatty Acids from New Zealand Milk and Bulk Milk Powder. J. Agric. Food Chem..

[175] Chesson L.A., Valenzuela L.O., O’Grady S.P., Cerling T.E., Ehleringer J.R. (2010). Hydrogen and Oxygen Stable Isotope Ratios of Milk in the United States. J. Agric. Food Chem..

[176] Altieri S., Saiano K., Biondi M., Ricci P., Lubritto C. (2020). Traceability of ‘Mozzarella Di Bufala Campana’ Production Chain by Means of Carbon, Nitrogen and Oxygen Stable Isotope Ratios. J. Sci. Food Agric..

[177] Silva A.V., Hélie J.F., de Andrade Caxito F., Monardes H., Mustafa A.F., Stevenson R. (2014). Multi-Stable Isotope Analysis as a Tool for Assessing the Geographic Provenance of Dairy Products: A Case Study Using Buffalo’s Milk and Cheese Samples from the Amazon Basin, Brazil. Int. Dairy J..

[178] Liu H., Zhao Q., Guo X., Tang C., Yu X. (2019). Application of Isotopic and Elemental Fingerprints in Identifying the Geographical Origin of Goat Milk in China. Food Chem..

[179] Mc Leod R.J., Prosser C.G., Wakefield J.W. (2016). Identification of Goat Milk Powder by Manufacturer Using Multiple Chemical Parameters. J. Dairy Sci..

